# A Comparative Transcriptomic Study Reveals Temporal and Genotype-Specific Defense Responses to *Botrytis cinerea* in Grapevine

**DOI:** 10.3390/jof11020124

**Published:** 2025-02-07

**Authors:** Flavia Angela Maria Maggiolini, Annalisa Prencipe, Carlo Bergamini, Antonio Domenico Marsico, Marco Vendemia, Marika Santamaria, Maria Angela Giannandrea, Margherita D’Amico, Lucia Rosaria Forleo, Rocco Perniola, Riccardo Velasco, Maria Francesca Cardone

**Affiliations:** 1 Council for Agricultural Research and Economics—Research Center Viticulture and Enology (CREA-VE), 8 Via Casamassima 148, 70010 Turi, Italy; 2 Department of Biosciences, Biotechnology and Environment, University of Bari “Aldo Moro”, 70125 Bari, Italy

**Keywords:** RNA-seq, gray mold, genotype-specific responses, metabolic flexibility, phenylpropanoid biosynthesis, *MAPK* signaling pathway

## Abstract

Grapevine (*Vitis vinifera* L.), a globally significant crop, is highly susceptible to *Botrytis cinerea*, the causative agent of gray mold disease. This study investigates transcriptomic responses to *B. cinerea* in tolerant and susceptible grapevine genotypes using RNA sequencing (RNA-seq). Differentially expressed genes (DEGs) were identified at three time points (T1, T2, T3), highlighting both genotype-independent and genotype-specific responses. Early-stage infection (T1) revealed rapid and robust activation of defense pathways in both genotypes, though the tolerant genotype showed enhanced modulation of metabolic processes by T2, prioritizing secondary metabolism and stress adaptation over growth. In contrast, the susceptible genotype exhibited less coordinated metabolic reprogramming, with delayed or weaker activation of key defense mechanisms. Gene Ontology and KEGG analyses identified critical pathways, including phenylpropanoid biosynthesis-like lignin metabolism, *MAPK* signaling, as well as candidate genes such as *WRKY* transcription factors and enzymes involved in cell wall fortification and antifungal compound biosynthesis. Genotype-specific responses emphasized metabolic flexibility as a determinant of resistance, with the tolerant genotype exhibiting superior resource allocation to defense pathways. These findings provide insights into the molecular basis of grapevine resistance to *B. cinerea*, offering potential targets for breeding or genetic engineering to enhance resilience and reduce fungicide dependency.

## 1. Introduction

The grapevine (*Vitis vinifera* L.) is one of the world’s most economically significant crops, central to the production of wine, table grapes, and raisins. However, its cultivation faces substantial challenges due to its susceptibility to fungal pathogens, with *Botrytis cinerea* Pers. Fr. being a major threat. *B. cinerea*, a necrotrophic fungus, causes grey mold disease, leading to significant pre- and post-harvest losses, especially in temperate and humid climates. Grapevine cultivars display varying levels of tolerance/susceptibility to *B. cinerea*, with some showing higher resilience due to their enhanced defense mechanisms. These differences in tolerance are primarily governed by complex molecular networks, including various defense signaling pathways, secondary metabolite production, and cell wall modifications [1]. Understanding the molecular mechanisms that confer tolerance to *B. cinerea* is crucial for developing more tolerant grapevine varieties, which reduces the need for fungicide applications and ensure sustainable grape production. To this end, examining the transcriptome of different grapevine cultivars using RNA sequencing (RNA-seq) has emerged as a powerful approach to identify key genes and pathways involved in host defense. In recent years, RNA-seq has become an invaluable tool for studying plant–pathogen interactions, as it allows for the high-throughput analysis of gene expression on a genome-wide scale. By comparing the transcriptomes of green and veraison berries, respectively resistant and susceptible to *B. cinerea* infections, researchers can identify differentially expressed genes (DEGs) that are involved in resistance mechanisms. These genes often belong to signaling pathways regulated by hormones such as jasmonic acid (JA), salicylic acid (SA), and ethylene (ET), which play central roles in modulating plant defense responses [2]. In grapevine, transcriptomic studies have identified several gene families and regulatory networks involved in defense responses. For example, transcription factors such as *WRKYs*, *MYBs*, and *NACs* play pivotal roles in orchestrating the expression of defense-related genes [3,4,5]. *WRKY* transcription factors, in particular, regulate in resistant Chinese wild grape (*Vitis amurensis* ‘Shoung You’) the activation of pathogenesis-related (PR) proteins, which are key components of the plant immune response [6,7]. Moreover, studies have highlighted the importance of genes involved in the phenylpropanoid pathway, which produces a variety of secondary metabolites with antifungal properties that contribute to the overall resistance of grapevines to necrotrophic pathogens. In particular, resistant grape berries at the phonological stage between bloom and veraison tend to upregulate these pathways more robustly and earlier than their susceptible counterparts after the veraison [2,8]. While much has been learned about the pathways involved in resistance, the timing of gene expression during infection remains a critical aspect of plant defense. In resistant cultivars, belonging to the species *V. amurensis*, genes involved in early defense responses are activated within hours of pathogen detection, leading to the rapid formation of physical barriers and the production of antimicrobial compounds. Conversely, susceptible *V. vinifera* cultivars often exhibit delayed or weak activation of these pathways, allowing the pathogen to establish a foothold and progress through the plant tissue. Temporal dynamics of gene expression are thus essential to understanding how plants cope with infection [4,9]. Investigating these temporal changes in grapevine could provide valuable insights into how specific genes and pathways influence the outcome of *B. cinerea* infection. In addition to hormonal signaling and secondary metabolism, structural changes in the cell wall are also pivotal in determining plant resistance to *B. cinerea*. The pathogen relies on a suite of enzymes to degrade plant cell walls, facilitating its entry into host tissues. In response, plants strengthen their cell walls by upregulating genes involved in lignin and callose biosynthesis, reinforcing the physical barrier to pathogen invasion [10]. Resistant grapevine organs, such as flowers, often show a more robust response in terms of cell wall fortification, which restricts pathogen penetration and reduces infection spread [9]. Understanding how the activation of cell wall-modifying genes contributes to resistance in grapevines could offer novel approaches for enhancing grapevine resilience through breeding or biotechnological interventions.

Our work aims to investigate the transcriptomic response of two *Vitis vinifera* genotypes, one susceptible and one more tolerant to *B. cinerea*, by performing RNA-seq at different time points after infection. The goal is to identify DEGs associated with resistance and to explore how the timing of their expression influences the susceptibility of the plant. Deciphering the temporal regulation of these genes could guide the development of strategies to enhance grapevine resistance, either through traditional breeding or biotechnological approaches, such as genetic engineering or genome editing. Moreover, this research aims to provide new insights into the genetic and molecular basis of resistance to *B. cinerea* in grapevines, paving the way for the development of more resilient grapevine cultivars and reducing the reliance on chemical treatments for disease management. Indeed, by understanding how the timing and intensity of gene expression affect the outcome of *B. cinerea* infection, we can design more effective strategies for mitigating the impact of this devastating pathogen on grape production.

## 2. Materials and Methods

### 2.1. Phenotypic Evaluation

Forty-one new table grape genotypes, obtained in our breeding program, were selected as they presented a bunch categorizable into the classes 5 (medium) to 9 (very dense) of the OIV descriptor n. 204. This descriptor categorizes the bunch compactness into one out of five categories, from 1 (very loose) to 9 (very dense), based on the number of visible pedicels and the mobility of the berries. For these genotypes, we further evaluated the bunch compactness, by using the CI12 index, proposed by Tello and Ibanez (2014), and their resistance to grey mold under field trials over two years without the use of chemical plant protection and/or resistance inductor. Briefly, the CI12 index is based on the combination of two easy-to-measure characteristics of the bunch (weight and length). Five bunches for each genotype were sampled at harvest time. The weight and the length of each bunch were determined using a precision scale.

The bunch degree of resistance to grey mold was evaluated by using the OIV descriptor n. 459 on all bunches present on five plants for each genotype before harvest. Five categories, from 1 (many wilted or rotten berries on all clusters) to 9 (only a few wilted or rotten berries on all bunches) were used (Figure S1A).

### 2.2. Bioassay in Controlled Conditions for Transcriptomic Analysis in Response to Botrytis cinerea

**Figure 1:**
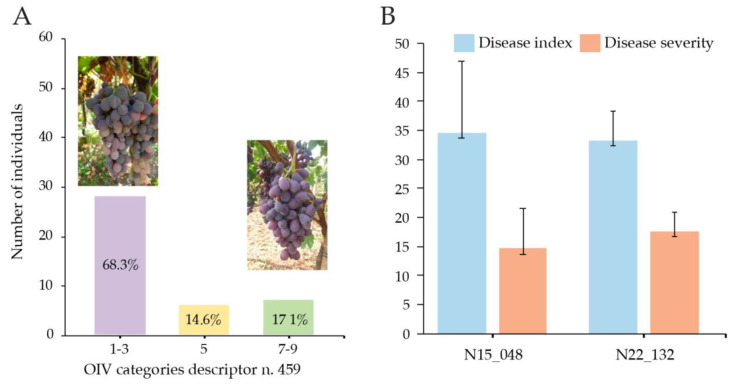
Evaluation of bunch compactness, resistance to grey mold, and responses to *B. cinerea* in new table grape genotypes: (**A**). Degree of resistance to grey mold of 41 genotypes, based on the categories reported in the OIV descriptor n. 459: 1–3 very little to little (many wilted or rotten berries on all clusters; some clusters can be slightly affected); 5 medium (large percentage of wilted or rotten clusters (up to 20%)—most clusters are moderately attacked, only few clusters are attacked more severely—drop off of clusters is only slight); 7–9: high to very high (only a few wilted or rotten berries on all clusters). Pictures above the histograms belong to N15/048 (category 1–3) and N22/132 (category 7–9), respectively. (**B**). Responses of the genotypes N15/048 and N22/132 to *B. cinerea* infections in bioassay performed in controlled conditions. Both the disease index and severity are presented as means of three replicates with standard deviation (vertical bars).

Two grape genotypes, N22/132 (‘Regal seedless’ x ‘Red globe’) and N15/048 (‘Cimminita’ x ‘Melissa’), were selected for this study based on previous field observations, which indicated differing responses to *B. cinerea* infection. Despite a similar degree of bunch compactness and a similar vegetative development, genotype N22/132 had been observed to exhibit greater tolerance, while variety N15/048 appeared more susceptible to the pathogen, according to the OIV descriptor n. 459 (Figure 1A).

Mature berries with pedicels were detached from three healthy bunches, harvested from the vine of the two genotypes, showing the same phenological stage, and then divided based on their content in total Solid Soluble, by flotation in different salt solutions. A total of 135 berries for each genotype were taken from the salt solution, corresponding to a soluble solid content between 14% and 15%. The surface of the berries was disinfected by dipping for 2 min in 2% (*w*/*v*) of sodium hypochlorite (NaClO) solution, rinsed with sterilized water, and then air-dried. Berries were artificially inoculated by dipping for 2 min in 5 L tanks containing a conidial suspension of *B. cinerea*. *B. cinerea* strain AS1, isolated from naturally infected berries and whose pathogenicity was evaluated by laboratory tests [11], and then used in this experiment. Conidia suspensions were prepared by removing the conidia from a 7-day-old culture with a sterile eyelet handle and then suspending in sterile water to the required concentration of 1 × 10^5^ conidia mL^−1^, which was estimated using a hemacytometer cell counting. Inoculated berries were placed in three boxes (L: 16.0 cm x W: 11.5 cm x H: 3.0 cm). One hundred thirty-five berries for each genotype were treated with sterilized water as a negative control. To create a humid environment, a wet paper was placed on the bottom of the boxes, which were individually covered face to face with a sterilized box. The two boxes were firmly wrapped together with Parafilm to prevent air leakage and incubated at 25 °C. The collection of samples was performed at four time points: prior to the inoculation (T0), 24 h (T1), 48 h (T2), and five days (T3) after inoculation. At each time and for each biological replicate, five berries were collected and peeled, and the skin was collected in a 1.5 mL centrifuge tube and immediately frozen in liquid nitrogen. Samples were ground into the tube with a pipette tip and the powder was stored at −80.

After five days of incubation at 25 °C, the disease severity (DS) was evaluated by using an empirical 0-to-4 rating scale, proposed by Marsico et al. [12]. Briefly, 0 = no visible symptoms; 1 = sporulation covering 5–10% of the berry surface; 2 = sporulation covering 10–25% of the berry surface; 3 = sporulation covering 25–50% of the berry surface; and 4 = sporulation covering more than 50% of the berry surface. The average disease severity in the two tested genotypes was calculated for each plastic box by using McKinney’s index [13], which provides a weighted measure of disease intensity by integrating the number of berries in each class with the severity of their symptoms. Furthermore, the average Disease Incidence (DI) was calculated as the percentage ratio between the number of infected berries and the total number of berries present in each plastic box. Collected data were analyzed using RStudio software (v.4.2.2) and Student’s *t*-test were used to detect differences between the response of the two table grape genotypes to *B. cinerea* infection.

### 2.3. RNA Extraction and Quantity and Quality Control

RNA extraction was performed as reported by Prencipe et al. (paper in press). Briefly, the sorbitol pre-wash step was performed with a buffer containing Tris-HCl, sorbitol, EDTA, PVP-40, and ß-mercaptoethanol to remove polysaccharides and polyphenols from macerated plant material. Samples were vortexed, centrifuged at 2500× *g*, and the supernatant discarded. RNA was then extracted using the Norgen Plant/Fungi Total RNA Purification Kit. RNA purity and yield were calculated using a Qubit 4 Fluorometer (Thermo Fisher Scientific, Waltham, MA, USA), while the 260/280 ratio was calculated using a NanoDrop 2000 Spectrophotometer (Thermo Fisher Scientific, Waltham, MA, USA). RNA integrity was assessed using the Agilent 2100 Bioanalyzer Instrument (Agilent, Santa Clara, CA, USA) that calculated the RIN values.

### 2.4. Library Preparation and RNA Sequencing

The RNA samples extracted from berries skin were redirected to the Novogene company (Cambridge, UK) for RNA-seq analysis. A total of 33 RNA-Seq libraries were constructed using the Eukaryotic mRNA-Seq (polyA enrichment, non-directional library). RNA library is formed by polyA capture and reverse transcription of cDNA. Library QC was performed. For each sample, the transcriptome sequencing was carried out by using Illumina NovaSeq X PE150 technology (Illumina Hayward, Hayward, CA, USA).

### 2.5. Differential Gene Expression Analysis and Function Enrichment Analysis

*V. vinifera* PN40024_v4 was used as a reference for read mapping and gene annotation, directly downloaded from the ENSEMBL repository (ensembl_56_vitis_vinifera_pn40024_v4_toplevel). Further, the gene annotation files were downloaded from the Novogene genome website directly (https://www.novogene.com/us-en/Reference/ReferenceForSpecies/index.html, accessed on 1 December 2024). The index of the reference genome was built using Hisat2 v2.0.5, and paired-end clean reads were aligned to the reference genome using Hisat2 v2.0.5. We selected Hisat2 [14] as the mapping tool because Hisat2 can generate a database of splice junctions based on the gene model annotation file and, thus, a better mapping result than other non-splice mapping tools. featureCounts v1.5.0-p3 [15] was used to count the read numbers mapped to each gene. And then FPKM of each gene was calculated based on the length of the gene and reads count mapped to this gene. Differential expression analysis was performed using DeSeq2 [16]. DESeq2 provides statistical routines for determining differential expression in digital gene expression data using a model based on the negative binomial distribution. The resulting *p*-values were adjusted using Benjamini and Hochberg’s approach for controlling the false discovery rate. Genes with an adjusted *p*-value ≤ 0.05 found by DESeq2 were assigned as differentially expressed.

Gene Ontology (GO) enrichment analysis and Kyoto Encyclopedia of Genes and Genomes (KEGG) pathway analysis were performed to identify functional and pathway-level associations for differentially expressed genes derived from RNA sequencing data. Differential expression was determined by comparing transcriptomic profiles of inoculated (I) and non-inoculated (NI) samples at multiple time points using a false discovery rate (FDR) threshold of <0.05 and a log2 fold-change cutoff of ±1.

For GO enrichment analysis, DEGs were categorized into biological processes (BP), molecular functions (MF), and cellular components (CC) using the Gene Ontology database. Enrichment analysis was conducted using Fisher’s exact test with FDR correction, and significantly enriched GO terms were identified based on a corrected *p*-value < 0.05.

For KEGG pathway enrichment analysis, DEGs were mapped to pathways in the KEGG database using the KEGG Automatic Annotation Server (KAAS). Enriched pathways were identified by comparing observed gene counts in each pathway to an expected distribution using a hypergeometric test. Pathways with an FDR-adjusted *p*-value < 0.05 were considered significantly enriched. Visualization of enriched GO terms and KEGG pathways was performed using R packages (v.4.2.2), including clusterProfiler and ggplot2, to provide an integrated view of genotype- and time-dependent transcriptional responses.

## 3. Results

### 3.1. Phenotypic Evaluation of 41 Genotypes and B. cinerea Infection of Berries

Since bunch compactness has a key role in botrytis infection [17], we evaluated this parameter in 41 new table grape genotypes. Among these, 39 out of 41 selected genotypes did not show significant differences in terms of bunch compactness, assessed by the CI12 index. Only two (N20/006 and N23/127) showed a significantly lower compactness index (Figure S1B). Differentiated responses regarding the degree of tolerance/susceptibility to grey mold were detected within the considered genotypes. Specifically, field observations carried out in two different years showed that about 68.0% of analyzed genotypes can be ascribed to the categories 1–3 (very little to little degree of tolerance to grey mold), about 15.0% to the category 5 (medium degree of tolerance to grey mold), and about 17.0% to the categories 7–9 (high to very high degree of tolerance to grey mold) (Figure 1A). To investigate the genotypic response to *Botrytis* infection, we randomly selected two individuals from the evaluated group with compact bunches that exhibited opposite infection responses: N22/132 (category 7–9) and N15/048 (category 1–3) Figure 1A. These selections were based on their consistent behavior across all field observations.

To further investigate the genotypic differences in response to *B. cinerea* infection observed in field conditions, a bioassay was conducted under controlled conditions (see Methods). This experiment aimed to validate the contrasting responses of the two selected genotypes, N22/132 and N15/048, previously identified as representative of opposite infection behavior based on field observations. Despite their contrasting behavior under field conditions, the controlled conditions did not replicate this divergence, and the results of the in vitro bioassay indicated no statistically significant differences between the two genotypes in terms of both disease severity and disease incidence (Figure 1B), suggesting that the in vitro infection conditions differ from the natural infection.

### 3.2. Transcriptomic Analysis of Grapevine Berries in Response to Botrytis cinerea

RNA sequencing (RNA-seq) was conducted to investigate the transcriptomic responses of two grapevine genotypes (N22/132 and N15/048) to *B. cinerea* infection. For each genotype, RNA was extracted from the berry skin at four time points: T0 (pre-inoculation), T1 (one day post-inoculation (dpi); both inoculated (I) and non-inoculated (NI) samples), T2 (two dpi; both I and NI), and T3 (five dpi; both I and NI). Three biological replicates were included for each time point and treatment, ensuring robust data for statistical comparisons.

RNA from grape berries was processed for RNA-seq, and 33 cDNA libraries were sequenced using the Illumina NovaSeq platform with paired-end 150 bp reads, yielding an average of 56.7 million raw reads per library. After quality control, 95.7% of reads were clean, with Q20 scores ranging from 96.66% to 97.24% and Q30 scores from 90.40% to 93.52%, confirming high data quality. Reads were aligned to the *Vitis vinifera* reference genome (PN40024_v4) and the expression level for each gene, in each sample was determined (Table S1). Based on the gene expression profile in each sample, Principal Component Analysis (PCA) was further performed, highlighting the separation between tolerant and susceptible genotypes along both PC1 (34.83%) and PC2 (15.22%), highlighting genotype-specific transcriptomic variation (Figure S2).

### 3.3. Gene Expression Profiles in Response to B. cinerea Inoculation at T1 and T2 in the Two Genotypes

Significant transcriptomic changes were detected in both genotypes over the course of infection. To investigate the transcriptomic responses of the two genotypes to *B. cinerea* infection for each genotype, at each time point, we searched for differentially expressed genes (DEGs) between the inoculated (I) sample in comparison to the non-inoculated sample (NI). In variety N15/048 (susceptible genotype), 1110 DEGs were identified in response to inoculation at T1, with 498 upregulated and 612 downregulated genes compared to the corresponding NI controls. At T2, the numbers decreased to 309 genes upregulated, and 195 downregulated. Unfortunately, T3 data are not available for this genotype since the RNA extraction from inoculated samples at T3 failed. By contrast, in variety N22/132 (tolerant genotype), the response at T1 involved 1415 upregulated and 1505 downregulated genes, indicating a broader and more extensive gene expression regulation. At T2, 249 genes were upregulated and 799 were downregulated, while at T3, the DEG count reached 310 upregulated and 887 downregulated genes (Figure S3). Considering the lack of T3 data of the susceptible genotype, we further compared the transcriptomic responses of the two genotypes to *B. cinerea* inoculation at T1 and T2, focusing on DEGs that maintained consistent expression profiles across the T1 and T2 time points. In the tolerant genotype (N22/132), 113 DEGs were upregulated at both T1 and T2, while 167 DEGs were consistently downregulated. Additionally, seven DEGs exhibited a switch in expression pattern over time, with two transitioning from upregulated at T1 to downregulated at T2, and five showing the reverse trend. In contrast, the susceptible genotype (N15/048) displayed a more restrained response, with only 98 DEGs consistently upregulated and 14 consistently downregulated at T1 and T2. A total of six DEGs switched expression patterns, with two transitioning from up to down and four from down to up.

### 3.4. Consistency and Divergence in Gene Expression Profiles Between Genotypes Over Time

To further investigate the genotype-dependent response to fungal infection, we compared DEGs at T1 and T2 between the tolerant and susceptible genotypes. Interestingly, we found that most of the DEGs are genotype-specific, and at T1, the number of DEGs up- and downregulated are almost equivalent, while at T2 the tolerant genotype exhibited more downregulated genes than upregulated in a ratio of 3:1. On the contrary the susceptible genotype exhibited only upregulated genes at T2 (Tables S2 and S3, Figure S4A,B).

**Figure 2:**
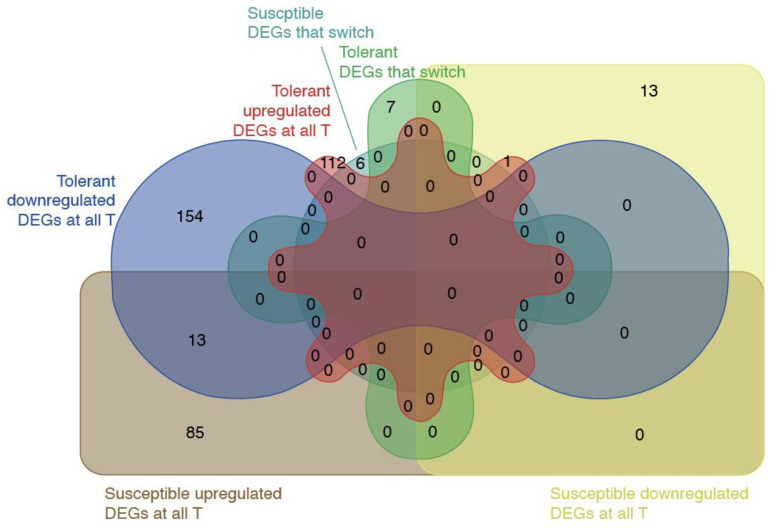
Venn diagram of DEGs with contrasting regulation between genotypes. The Venn diagram highlights 14 DEGs (13 + 1) shared between the tolerant and susceptible genotypes that exhibited consistent expression profiles across T1 and T2 within each genotype but displayed contrasting regulation patterns between the genotypes.

At T1, 113 DEGs were shared between the two genotypes, exhibiting the same regulation pattern in 94 downregulated and 19 upregulated genes. Additionally, 146 DEGs were common to both genotypes but displayed opposite regulation patterns: 79 genes were downregulated in the tolerant genotype and upregulated in the susceptible one, while the remaining 67 showed the reverse trend (Table S2, Figure S4A). By T2, the number of shared DEGs with consistent regulation across genotypes dropped to eight, including seven downregulated and one upregulated gene. Notably, among the eight consistently regulated DEGs at T2, six were not differentially expressed at T1 in either genotype. Two genes, Vitvi02g00230 and Vitvi12g04561, were identified as DEGs already at T1 but only in the tolerant genotype. Moreover, 109 DEGs were found to switch regulation between genotypes, with 106 genes downregulated in the tolerant genotype but upregulated in the susceptible one, and 3 genes showed the opposite behavior (Table S3, Figure S4B). In a comparison of these results using Venn diagrams, we identified 14 DEGs shared between the two genotypes that exhibited the same expression profile along time points within each genotype but contrasting regulation patterns between the genotypes (Figure 2, Table S4). These genes represent a subset of interest, as they may play key roles in defining the differential responses to *B. cinerea* infection observed between the tolerant and susceptible genotypes. Further functional characterization of these genes could provide insights into genotype-specific mechanisms underlying disease resistance and susceptibility.

### 3.5. Gene Ontology Enrichment Analysis

Gene Ontology (GO) enrichment analysis was performed to characterize the functional categories associated with DEGs between inoculated and non-inoculated samples at each time point for the two genotypes. At T1, in the tolerant genotype, downregulated DEGs (SData1, Figures S5A and S6A) were enriched in biological processes (BPs) such as peptidyl-tyrosine phosphorylation (GO:0018108), detoxification (GO:0098754), and photosynthesis (GO:0015979). Cellular components (CCs) included cytosolic ribosome (GO:0022626), thylakoid (GO:0009579), and extracellular region (GO:0005576), with molecular functions (MFs) like protein tyrosine kinase activity (GO:0004713) and glutathione transferase activity (GO:0004364). In the susceptible genotype, downregulated DEGs (SData1, Figures S5B and S6B) were strongly linked to photosynthesis-related terms (GO:0015979) in BPs, thylakoid and photosystem components (e.g., GO:0009579, GO:0009522), and heme binding (GO:0020037) in MFs. Upregulated DEGs in tolerant genotypes (SData1, Figures S5A and S6C) were associated with chromatin organization, including the DNA packaging complex (GO:0044815) and structural chromatin constituents (GO:0030527). In the susceptible genotype, instead, upregulated DEGs (SData1, Figures S5B and S6D) included terms related to photosynthesis (GO:0015979), flavonoid biosynthesis (GO:0009813), and heat response (GO:0009408) in BPs, with plastid and thylakoid components (GO:0044435, GO:0009535) enriched in CCs.

Considering T2, we found that in the tolerant genotype, downregulated DEGs (SData1, Figure S7A and S8A) were significantly enriched in biological processes (BPs) related to secondary metabolism (e.g., GO:0044550, secondary metabolite biosynthetic process), phenylpropanoid biosynthesis (GO:0009699), response to ethylene (GO:0009723), and lignin metabolic processes (GO:0009808). Cellular components (CCs) included extracellular regions (GO:0005576) and COPI vesicle structures (e.g., GO:0030137), while molecular functions (MFs) were associated with enzymatic activities, including chitinase (GO:0004568) and peroxidase (GO:0004601). On the contrary, the susceptible genotype downregulated DEGs (SData1, Figures S7B and S8B) showed limited enrichment, with terms like extracellular region (GO:0005576) and monooxygenase activity (GO:0004497). For the tolerant genotype, upregulated DEGs (SData1, Figure S7A and S8C) were enriched in transmembrane transporter activities, including oligopeptide transporters (GO:0035673) and amide transporters (GO:0042887). In the susceptible genotype, upregulated DEGs (SData1, Figure S7B and S8D) highlighted processes such as polyketide metabolism (GO:0030638), ethylene response (GO:0009723), and metal ion transport (GO:0030001). Enriched CCs included extracellular regions (GO:0005576), while MFs involved transferase activities (e.g., GO:0016747) and glutathione transferase activity (GO:0004364). These results underscore genotype- and inoculation-specific regulatory patterns in metabolic pathways, stress responses, and transport processes.

For the tolerant genotype, GO enrichment was also performed at the T3. The analysis revealed distinct functional patterns between downregulated and upregulated DEGs when comparing inoculated versus non-inoculated samples. Downregulated DEGs (SData1, Figure S9A) were predominantly associated with processes linked to stress responses and secondary metabolism, such as secondary metabolite biosynthetic processes (GO:0044550, GeneRatio: 19/530), phenylpropanoid metabolic processes (GO:0009698, GeneRatio: 12/530), and response to ethylene (GO:0009723, GeneRatio: 11/530). These genes also contributed to detoxification pathways (GO:0098754, GeneRatio: 19/530), lignin metabolism (GO:0009808, GeneRatio: 7/530), and hydrogen peroxide catabolism (GO:0042744). Enriched molecular functions included transferase activities (e.g., GO:0016746), calcium ion binding (GO:0005509), and peroxidase activity (GO:0004601). Cellular components were notably enriched in the extracellular region (GO:0005576). Conversely, upregulated DEGs (SData1, Figure S9B) were strongly enriched in photosynthesis-related processes (GO:0015979) and flavonoid biosynthesis (GO:0009813), indicating a shift toward energy production and secondary metabolite accumulation. These genes were localized in plastid-associated compartments, including thylakoid membranes (GO:0009535) and photosystems (GO:0009521), highlighting a chloroplast-specific upregulation in response to inoculation.

### 3.6. KEGG Enrichment Analysis of Differentially Expressed Genes in Tolerant and Susceptible Genotypes

**Figure 3:**
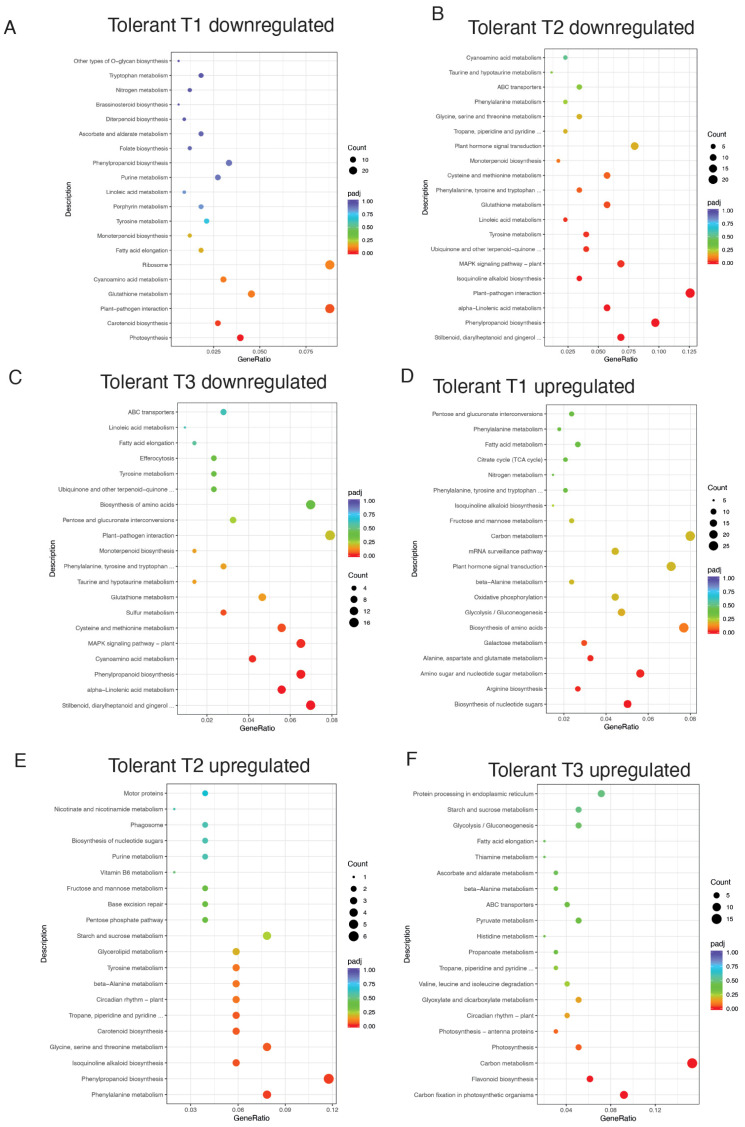
KEGG analysis of pathways enriched in the tolerant genotype in response to Botrytis infection: (**A**). KEGG enrichment of DEGs downregulated at T1. (**B**). KEGG enrichment of DEGs downregulated at T2. (**C**). KEGG enrichment of DEGs downregulated at T3. (**D**). KEGG enrichment of DEGs upregulated at T1. (**E**). KEGG enrichment of DEGs upregulated at T2. (**F**). KEGG enrichment of DEGs upregulated at T3.

KEGG enrichment analysis revealed few enriched pathways with respect to the GO enrichment analysis. We found distinct pathway modulations in tolerant and susceptible genotypes at different time points post-inoculation compared to non-inoculated samples. In the tolerant genotype, downregulated DEGs at T1 (SData2, Figure 3A) were significantly enriched in pathways related to photosynthesis (vvi00195), carotenoid biosynthesis (vvi00906), and plant–pathogen interaction (vvi04626), suggesting early suppression of primary metabolic and defensive interactions. By T2 (SData2, Figure 3B), additional pathways emerged, including phenylpropanoid biosynthesis (vvi00940), *MAPK* signaling (vvi04016), and glutathione metabolism (vvi00480), reflecting a shift toward oxidative stress management and secondary metabolism. At T3 (SData2, Figure 3C), pathways associated with secondary metabolites, such as stilbenoid biosynthesis (vvi00945) and sulfur metabolism (vvi00920), dominated, highlighting a continued focus on stress adaptation and detoxification processes.

Conversely, upregulated DEGs in the tolerant genotype at T1 (SData2, Figure 3D) involved pathways such as nucleotide sugar metabolism (vvi01250) and amino acid metabolism (vvi00220), pointing to enhanced biosynthetic and metabolic readiness. At T2 (SData2, Figure 3E), phenylalanine and phenylpropanoid biosynthesis (vvi00360, vvi00940) gained prominence, while T3 (SData2, Figure 3F) emphasized carbon fixation (vvi00710) and flavonoid biosynthesis (vvi00941), indicating a transition toward energy production and secondary metabolite accumulation.

**Figure 4:**
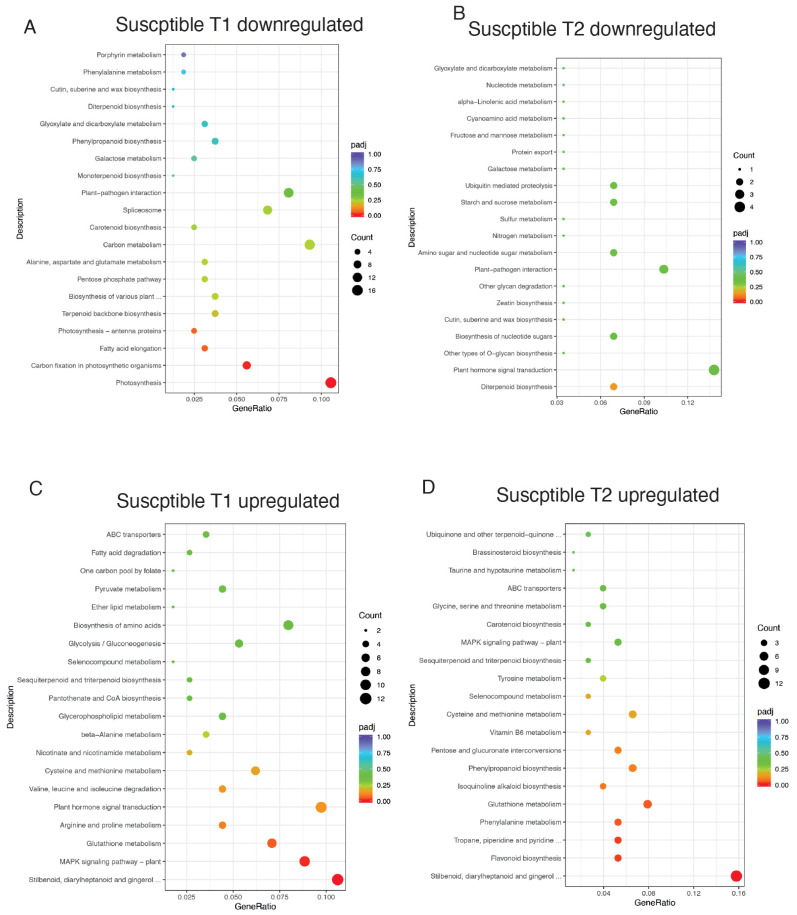
KEGG analysis of pathways enriched in the susceptible genotype in response to Botrytis infection: (**A**). KEGG enrichment of DEGs downregulated at T1. (**B**). KEGG enrichment of DEGs downregulated at T2. (**C**). KEGG enrichment of DEGs upregulated at T1. (**D**). KEGG enrichment of DEGs upregulated at T2.

The susceptible genotype displayed contrasting patterns. At T1 (SData2, Figure 4A), downregulated DEGs were enriched in photosynthesis-related pathways (vvi00195) and carbon fixation (vvi00710), indicative of impaired photosynthetic capacity. By T2 (SData2, Figure 4B), diminished activity in starch and sucrose metabolism (vvi00500) and plant hormone signaling (vvi04075) highlighted reduced metabolic coordination. Upregulated DEGs in the susceptible genotype at T1 (SData2, Figure 4C) and T2 (SData2, Figure 4D) revealed consistent activation of pathways like stilbenoid biosynthesis (vvi00945), *MAPK* signaling (vvi04016), and glutathione metabolism (vvi00480), reflecting a reactive stress response dominated by secondary metabolism and oxidative defense.

### 3.7. Candidate Genes Underlying Genotype-Independent and Genotype-Dependent Responses to Botrytis Infection

Considering both the comparison between DEGs in the two genotypes over time and the enrichment analyses, we selected candidate genes by focusing on two primary criteria. First, we identified 121 DEGs that exhibited consistent expression patterns across both genotypes at T1 (113 DEGs) and T2 (8 DEGs) in inoculated versus non-inoculated samples (Table S4 and Figure S3A,B), representing a genotype-independent response to *Botrytis* infection. For example, genes involved in photosynthesis (e.g., Vitvi12g00475, GO:0015979) and glutathione metabolism (e.g., Vitvi19g04456, pathway vvi00480) were consistently downregulated in both genotypes at T1, highlighting their role in general stress responses. Genes like Vitvi04g01220 (pathway vvi04626, plant–pathogen interaction) and Vitvi04g00115 (pathway vvi04016, *MAPK* signaling) also showed consistent regulation across genotypes, emphasizing the importance of defensive signaling pathways in early infection stages.

**Figure 5:**
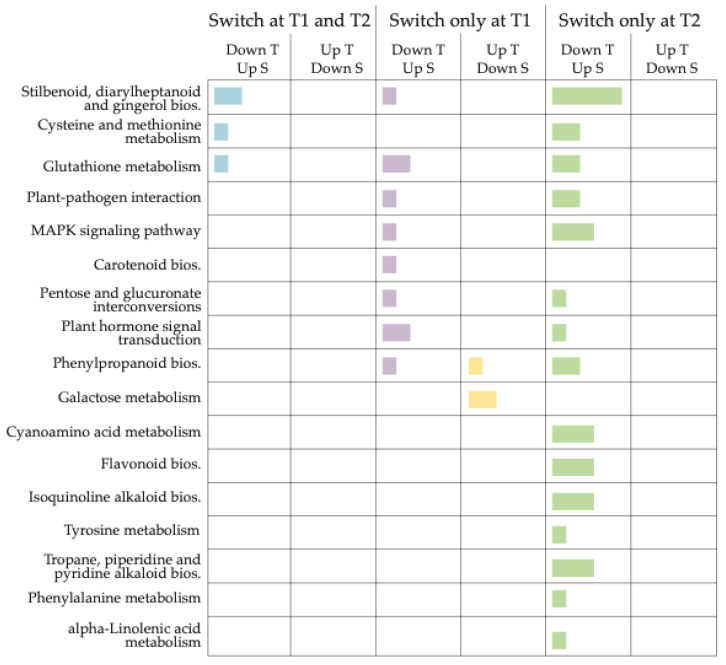
Enriched KEGG pathways of candidate DEGs with expression profile shifts between tolerant and susceptible genotypes at T1 and T2 (Table S6). The figure shows enriched pathways belonging to DEGs that switch their expression profile across the two genotypes at T1 and/or T2. A bar in the “Switch at T1 and T2” column indicates a consistent expression switch at both time points, while bars in “Switch only at T1” or “Switch only at T2” denote switches occurring exclusively at those time points. If a pathway has bars in both “Switch only at T1” and “Switch only at T2”, but not in “Switch at T1 and T2”, it means different DEGs within the same pathway underwent expression switching at each time point. The size of the colored bars indicates the proportion of DEGs associated with each enriched KEGG pathway that were either upregulated or downregulated in tolerant (T) or susceptible (S) genotypes.

Second, we focused on DEGs with contrasting expression patterns between tolerant and susceptible genotypes in at least one time point or showing consistent trends within each genotype across T1 and T2 (Table S6, Figure 2). These genes represent genotype-dependent responses to *Botrytis* infection. Notable examples include Vitvi16g01501 (vvi00480, glutathione metabolism), which switched expression between genotypes, highlighting its role in oxidative stress responses and detoxification pathways. Genes involved in key signaling processes, such as Vitvi01g01803 (vvi04626, plant–pathogen interaction; vvi04016, *MAPK* signaling) and Vitvi08g00793 (vvi04016, *MAPK* signaling; vvi04626, plant-pathogen interaction), also exhibited contrasting expression patterns, emphasizing their potential role in mediating genotype-specific defense mechanisms. Additionally, genes related to secondary metabolite biosynthesis, such as Vitvi00g04407, Vitvi16g04347, and Vitvi10g04623 (vvi00945, stilbenoid biosynthesis), demonstrated significant genotype-specific metabolic shifts. Other examples include Vitvi12g00445 (vvi00270, cysteine, and methionine metabolism) and Vitvi05g00715 (vvi04016, *MAPK* signaling; vvi04075, plant hormone signal transduction), which may further reflect differential responses to biotic stress. Additionally, pathways related to secondary metabolites and signaling include genes such as Vitvi05g00715 (vvi04016, *MAPK* signaling; vvi04075, plant hormone signal transduction) and Vitvi18g00362 (pathways vvi00950, isoquinoline alkaloid biosynthesis; vvi00350, tyrosine metabolism; vvi00360, tropane, piperidine, and pyridine alkaloid biosynthesis; vvi00960, phenylalanine metabolism). Considering the KEGG enrichment of these candidate genes, we highlighted the dynamic temporal regulation of various metabolic and signaling pathways, with distinct shifts occurring at T1 and T2 (Figure 5). Notably, more pathways exhibit significant changes at T2, suggesting a stronger or delayed response at this time point. Pathways related to stress responses, metabolism, and signaling are differentially regulated, indicating time-dependent biological adjustments. The data suggest that metabolic adaptation occurs progressively, with some pathways responding early (T1) while others shift later (T2).

## 4. Discussion

Understanding the molecular basis of grapevine resistance to *B. cinerea* is critical for developing effective strategies to mitigate the impact of this devastating pathogen. Grapevine cultivars exhibit varying responses to infection, influenced by complex transcriptional and metabolic networks [6,18,19]. Our study investigates these responses by examining and comparing the transcriptomes of a tolerant and a susceptible genotype over multiple time points in response to artificial fungal inoculum, aiming at identifying key genes and pathways involved in the defense against *B. cinerea*. The analysis provides valuable insights into the temporal and genotype-specific dynamics of the grapevine’s defense mechanisms. Indeed, the expression profiles of DEGs reveal early, genotype-dependent responses, with a higher number of DEGs identified at the first time point (T1) compared to T2 and T3, reflecting the rapid activation of defense mechanisms during the early stages of infection (Figure S3). This trend is consistent with the idea that plants mount a fast and robust response to pathogen invasion, which is critical for limiting pathogen spread and severity of infection [6,20,21]. Interestingly, the majority of DEGs identified through Venn analysis were genotype-specific (Figure S4A,B), indicating that the two genotypes, tolerant (N22/132) and susceptible (N15/048), exhibit distinct molecular responses.

At T1, both the tolerant and susceptible genotypes showed an equal distribution of upregulated and downregulated DEGs, suggesting an initial broad-scale activation of defense pathways in both genotypes (Table S2, Figure S4A). However, by T2, the tolerant genotype showed a significant shift, with a much higher number of downregulated DEGs (a ratio of approximately 3:1), contrasting with the susceptible genotype, which exhibited only upregulated genes at this stage (Table S3, Figure S4B). This discrepancy highlights a key difference between the two genotypes: the tolerant genotype may be actively suppressing certain metabolic processes, possibly redirecting resources toward defense-related functions such as secondary metabolism and stress response, while the susceptible genotype shows a more unidirectional activation of genes, indicating a lack of coordinated regulation between growth and defense processes. Interestingly, genotype-dependent responses against *B. cinerea* and other fungal pathogens have been documented in various plant species [6,18,19,22,23]. Moreover, variety-dependent transcriptomic profiles motivated in a time-dependent mode have been described in grapevine also as a defense mechanism against *Aspergillus carbonarius* infections [24], underlying the importance of genetic factors in plant defense against pathogen attacks.

Looking at the functional annotation of DEGs between the two genotypes allowed us to infer new clues about the responses to *B. cinerea* in grapevine, especially regarding the genotype-dependent responses. The GO enrichment analysis revealed distinct functional categories associated with the DEGs in both genotypes. In the tolerant genotype, downregulated DEGs at T2 were significantly enriched in stress-responsive and metabolic processes, including secondary metabolite biosynthesis (GO:0044550), phenylpropanoid biosynthesis (GO:0009699), and lignin metabolic processes (GO:0009808), which are critical components of plant defense mechanisms [23,24,25,26,27]. The enrichment of molecular functions such as peroxidase activity (GO:0004601) and chitinase (GO:0004568) further supports the involvement of defense-related enzymes in the response to infection (SData1, Figure S8A). Indeed, both peroxidases and laccases are involved in the polymerization of lignin monomers, contributing to cell wall strengthening during pathogen attack [23,26]. While peroxidases generate radicals for lignin cross-linking, laccases also play a crucial role in lignin biosynthesis and modification, particularly in grapevine [23,28,29]. Chitinases degrade chitin, a major component of fungal cell walls, thereby inhibiting fungal growth and providing a direct defense mechanism against fungal pathogens [30]. These findings suggest that the tolerant genotype tries to balance growth and defense, actively suppressing certain growth-related pathways in favor of reinforcing defense responses. This is also consistent with the upregulation, in the tolerant genotype, of DEGs linked to transport processes, indicating the mobilization of resources essential for sustaining defense responses [23,31]. In contrast, the susceptible genotype showed limited enrichment in these functional categories, with only a modest response in terms of extracellular region components (GO:0005576) and monooxygenase activity (GO:0004497) (SData1, Figure S8B). Moreover, the susceptible genotype consistently displayed a stronger association of downregulated DEGs with photosynthesis-related terms and thylakoid structures, indicating a compromised photosynthetic capacity under stress conditions [32]. Upregulated DEGs in this genotype emphasized secondary metabolism and metal ion transport (SData1, Figure S8D), which may indicate reactive stress pathways that are less coordinated than those in the tolerant genotype, potentially contributing to increased vulnerability to pathogen attack [33].

**Figure 6:**
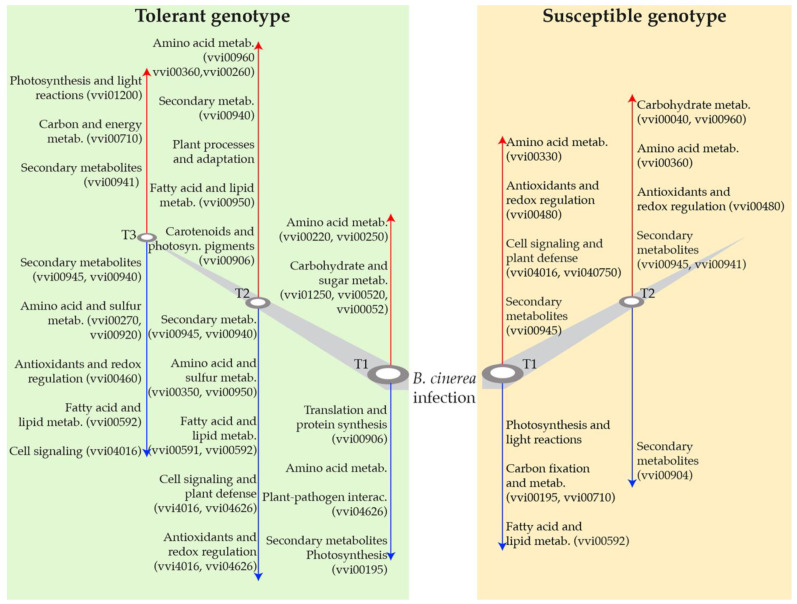
Summary of pathway activation in tolerant and susceptible genotypes. The figure highlights the pathways that are up- or downregulated in both genotypes following infection over time.

The KEGG enrichment analysis (SData2, Figure 3, Figure 4 and Figure 5) further supports the findings from the GO analysis, suggesting, in our opinion, that the tolerant genotype is able to modulate its metabolism more effectively in response to *B. cinerea* infection, prioritizing defense and repair mechanisms over growth, whereas the susceptible genotype may be unable to efficiently reprogram its metabolism to adapt to stress (Figure 6). Indeed the tolerant genotype exhibited an early resource allocation shift from primary metabolism toward defense, consistent with the “growth–defense trade-off” theory in stress responses [34,35], followed by a clear shift toward pathways involved in secondary metabolism, such as phenylpropanoid biosynthesis (vvi00940) and stilbenoid biosynthesis (vvi00945) (Figure S10), reflecting a sustained focus on stress adaptation and detoxification, during which secondary metabolism plays a fundamental role in long-term defense strategies [18,36] (SData2, Figure 3 and Figure 6). In contrast, the susceptible genotype showed a stronger association with pathways related to photosynthesis (vvi00195) and carbon fixation (vvi00710), indicating a compromised ability to effectively modulate metabolic processes in response to infection (SData2, Figure 4 and Figure 6), supporting that stress-induced impairment of energy production is a hallmark of reduced tolerance [37].

Moreover, in the susceptible genotype upregulated pathways were predominantly related to oxidative stress responses, such as glutathione metabolism (vvi00480), while limited activation of defense-related pathways, such as starch and sucrose metabolism (vvi00500) and plant hormone signaling (vvi04075), were observed, suggesting inadequate metabolic reprogramming to counter stress and a more reactive, rather than proactive, response to pathogen invasion (SData2) [33,37]. By looking at DEGs in these pathways we were able to find interesting candidate genes not only related to the common defense responses to *B. cinerea* in grapevine (Table S5), but mostly important involved in the different abilities of tolerant and susceptible genotypes to respond to the fungal infection at an early stage (Table S6). Specifically, looking at DEGs consistently regulated across both tolerant and susceptible genotypes at T1 and T2, we were able to identify candidate genes involved in the previously mentioned pathways (Table S5). Downregulated genes such as Vitvi12g00475 (photosynthesis, GO:0015979) and Vitvi19g04456 (glutathione metabolism, vvi00480) highlight conserved stress responses involving reduced energy expenditure and oxidative stress management, consistent with findings on redox regulation in plants [33,38]. Defense signaling pathways, including Vitvi04g01220 (plant–pathogen interaction, vvi04626) and Vitvi04g00115 (*MAPK* signaling, vvi04016) (Figure S11), also displayed consistent regulation, emphasizing their universal roles in early infection stages [39]. In contrast, genotype-dependent responses were revealed by DEGs with contrasting regulation patterns between the genotypes (Table S6). Notably, mostly these genes belong to the same pathways with respect to those involved in the genotype-independent responses. This finding further underscores the complexity of genotype-specific regulatory networks and highlights that metabolic flexibility and pathway prioritization are key determinants of resistance [27,40]. For example, Vitvi16g01501 (glutathione metabolism, vvi00480) exhibited genotype-specific shifts in regulation, indicating its role in differential stress adaptation [41]. Indeed glutathione metabolism participates in maintaining redox balance during pathogen attack [42]. Genes associated with stilbenoid biosynthesis (vvi00945) (Figure S10), such as Vitvi00g04407, Vitvi16g04347, and Vitvi10g04623, showed significant genotype-specific metabolic shifts, reinforcing their role in secondary metabolite-mediated defense. Additionally, genes involved in signaling pathways and secondary metabolism also displayed genotype-specific expression. For example, Vitvi01g01803 and Vitvi08g00793 were differentially regulated between genotypes in both the plant–pathogen interaction (vvi04626) and *MAPK* signaling pathways (vvi04016), suggesting their involvement in mediating genotype-specific defense responses. Also, the genes Vitvi05g00715 Vitvi04g00115 (Figure S11) with contrasting regulated in the tolerant genotype, are part of the *MAPK* signaling pathway (vvi04016), which transduces stress signals leading to defense responses (Figure S11) [39,43].

Moreover, other metabolic and signaling pathways were dynamically regulated, with key genes such as Vitvi18g00362 (isoquinoline alkaloid biosynthesis, vvi00950; tyrosine metabolism, vvi00350; tropane, piperidine, and pyridine alkaloid biosynthesis, vvi00360; phenylalanine metabolism, vvi00960) [44] exhibiting genotype-dependent responses. These findings suggest that metabolic and signaling networks are tightly regulated and dynamically adjusted based on genotype, likely contributing to differential resistance to *Botrytis* infection.

Our analysis, also, identified genes from prominent transcription factor (TF) families, including *ERF*, *MYB*, *NAC*, and *WRKY*, among DEGs across stress–response categories. TFs consistently regulated in both tolerant and susceptible genotypes highlight a role in genotype-independent stress responses, likely reflecting universal mechanisms of resilience. Meanwhile, TFs showing contrasting regulation patterns between genotypes, but consistent trends within each genotype across T1 and T2, suggest involvement in genotype-dependent pathways tailored to specific genetic backgrounds. Recent studies have emphasized the significance of *ERF* and *MYB* families in regulating responses to biotic stress with particular referment to *B. cinerea* and fungal infection, including the regulation of defense genes via *JA/ET* pathways [7,18,31]. *WRKY* and *NAC* families are similarly implicated in transcriptional reprogramming under stress [7]. The dual regulation patterns observed here, alongside consistent stress-resistance trends in past research [31], underscore their functional versatility and critical roles in plant stress adaptation.

These findings are consistent with previous studies suggesting that specific genes, may have contrasting effects on pathogen resistance depending on the genotype [31]. The identification of these genes could provide valuable insights into the molecular basis of differential susceptibility and resistance in grapevines, and their further functional validation could help to develop new tools for breeding porpoises including marker-assisted selection and editing, and thus refine strategies to enhance crop resilience.

## 5. Conclusions

Overall, our analysis sheds light on the interplay of conserved and genotype-specific mechanisms, providing a valuable foundation for the functional validation and development of disease-resistant grapevine cultivars. The differences observed between the two genotypes underscore the complexity of plant responses to biotic stress, with the tolerant genotype exhibiting a more coordinated and robust defense response that effectively limits pathogen spread. In contrast, the susceptible genotype’s inability to efficiently modulate its metabolic networks likely contributes to its higher susceptibility to *B. cinerea*. These results suggest that metabolic flexibility and pathway prioritization are essential for biotic stress resilience and may offer potential targets for improving disease resistance in grapevines. Furthermore, the identification of candidate genes that exhibit contrasting regulation in the two genotypes provides valuable insights into the molecular mechanisms underlying resistance and susceptibility, paving the way for future research aimed at developing grapevine cultivars with enhanced resistance to *B. cinerea*.

Notably, even if in the in vitro bioassay the differences observed between the two genotypes were not statistically significant, in the field, N22/132 consistently showed higher tolerance. This discrepancy may be due to the higher aggressive condition of the artificial inoculum with respect to the natural infection, supporting the importance of studying the molecular machinery involved in the defense response. Also, metagenomic analyses on the berry-associated microbiome are ongoing, and they will provide crucial insights into its composition, elucidating the microbiome’s putative role in genotype-specific disease tolerance.

In conclusion, the genotype-specific regulation of gene expression in response to *B. cinerea* infection is a key factor in determining the outcome of infection. By focusing on early responses and identifying genes with consistent expression patterns, this study provides valuable insights into the complex molecular networks that govern plant defense. The contrasting responses observed between the tolerant and susceptible genotypes emphasize the importance of metabolic reprogramming and coordinated stress responses in determining resistance to biotic stress. Further research into the functional roles of the identified candidate genes will be critical for understanding the genetic basis of resistance and for developing more resilient grapevine varieties.

## Data Availability

Raw sequencing data from Illumina sequencing experiments from this study have been submitted to the Sequence Read Archive (SRA; https://www.ncbi.nlm.nih.gov/sra/, accessed on 18 December 2024) under the BioProject ID PRJNA1211507.

## Supplementary Materials

The following supporting information can be downloaded at: https://www.mdpi.com/article/10.3390/jof11020124/s1, Figure S1. Assessment of resistance to grey mold and bunch compactness Index in new table grape genotypes A. Categories of the OIV descriptor n. 459—Cluster: degree of resistance to grey mold. 1 to 3 = many wilted or rotten berries on all clusters; some clusters can be slightly affected. Drop off of clusters. 5 = large percentage of wilted or rotten clusters (up to 20%)—most clusters are moderately attacked, only a few clusters are attacked more severely—drop off of clusters is only slight or no drop off at all. 7 to 9 = only a few wilted or rotten berries on all clusters (only little clusters are slightly attacked). No drop off of clusters. Evaluation of the appearance of grey mold on all clusters from at least 5 vines before harvest. B. Bunch compactness index (CI12) of 41 new table grape genotypes. Data are presented as the means of five replicates with standard deviation (vertical bars). * Columns labeled by different letters are significantly different according to Tukey’s test (*p* < 0.05). Figure S2. Principal component analysis (PCA) of gene expression profiles in tolerant and susceptible genotypes. The figure reports PCA analysis based on the gene expression profiles of each sample. The analysis shows a separation between tolerant and susceptible genotypes along PC1 (34.83%) and PC2 (15.22%). Each point represents an individual sample, with letters (A, B, and C) following T (tolerant genotype) or S (susceptible genotype) representing the biological replicates. NI = non-inoculated; T0, T1, T2, T3 = time points. Figure S3. DEGs histogram. The figure shows the total number of downregulated and upregulated DEGs in response to Botrytis infection (I vs. NI) for both tolerant and susceptible genotypes across the three time points considered. Figure S4. Venn diagrams for shared DEGs at T1 and T2. A. Venn diagram illustrating the shared and unique DEGs between the two genotypes at T1. B. Venn diagram illustrating the shared and unique DEGs between the two genotypes at T2. Figure S5. Up- and downregulated DEGs associated with enriched GO terms at T1. A. The bar charts display the number of DEGs associated with enriched GO terms in the tolerant genotype across three categories: biological processes (BP), cellular components (CC), and molecular functions (MF), highlighting genotype-specific patterns of gene regulation in response to stress at T1. B. The bar charts display the number of DEGs associated with enriched GO terms in the susceptible genotype across three categories: biological processes (BP), cellular components (CC), and molecular functions (MF), highlighting genotype-specific patterns of gene regulation in response to stress at T1. Figure S6. GO enrichment analysis of DEGs in response to Botrytis infection at T1. A. GO enrichment of DEGs downregulated in the tolerant genotype. B. GO enrichment of DEGs downregulated in the susceptible genotype. C. GO enrichment of DEGs upregulated in the tolerant genotype. D. GO enrichment of DEGs upregulated in the susceptible genotype. Figure S7. Up- and downregulated DEGs associated with enriched GO terms at T2. A. The bar charts display the number of DEGs associated with enriched GO terms in the tolerant genotype across three categories: biological processes (BP), cellular components (CC), and molecular functions (MF), highlighting genotype-specific patterns of gene regulation in response to stress at T2. B. The bar charts display the number of DEGs associated with enriched GO terms in the susceptible genotype across three categories: biological processes (BP), cellular components (CC), and molecular functions (MF), highlighting genotype-specific patterns of gene regulation in response to stress at T2. Figure S8. GO enrichment analysis of DEGs in response to Botrytis infection at T2. A. GO enrichment of DEGs downregulated in the tolerant genotype. B. GO enrichment of DEGs downregulated in the susceptible genotype. C. GO enrichment of DEGs upregulated in the tolerant genotype. D. GO enrichment of DEGs upregulated in the susceptible genotype. Figure S9. GO enrichment analysis of DEGs in response to Botrytis infection at T3. A. GO enrichment of DEGs downregulated in the tolerant genotype. B. GO enrichment of DEGs upregulated in the tolerant genotype. Figure S10. KEGG enrichment analysis of stilbenoid, diarylheptanoid, and gingerol biosynthesis pathway. The figure shows a comparison of up- and downregulated genes associated with the vvi00945 pathway, in both tolerant and susceptible genotypes after infection over time. Figure S11. KEGG enrichment analysis of *MAPK* signaling pathway. The figure shows a comparison of up- and downregulated genes associated with the vvi04016 pathway, in both tolerant and susceptible genotypes after infection over time. Table S1. Gene expression level for each gene and in each sample, as derived bu the calculation of FPKM (short for the expected number of Fragments Per Kilobase of transcript sequence per Millions base pairs sequenced), based on the abundance of transcripts(reads count), gene length and sequencing depth. The FPKM is the most common method of estimating gene expression levels, which takes the effects into consideration of both sequencing depth and gene length on counting of fragments; Table S2: List of DEGs resulting by the VENN comparison at T1. Name of comparison is reported in column A, number of DEGs included in each category in column B; Gene ID of the elements included in each caterogy is reported in column C; Table S2: List of DEGs resulting by the VENN comparison at T1. Name of comparison is reported in column A, number of DEGs included in each category in column B; Gene ID of the elements included in each caterogy is reported in column C; Table S4: List of 14 DEGs shared between the two genotypes that exhibited the same expression profile along time points within each genotype but contrasting regulation patterns between the genotypes; Table S5: List of Candidate DEGs releted to the common (non genotype-dependent) response to B.cinerea. Expression profile (TimePoint), is reported in columns B. The ratio of DEGs is also reported. Columns from C to I reported information about Gene Ontoloy and Pathway annotation for each gene, if avilable (na = not available); Table S6: List of Candidate DEGs that switch the expression profile among the two genotypes. Expression profile (TimePoint), is reported in columns A. Columns C and D reported information about the enriched Pathways annotation for each gene.
