# Supplementary material for: A Comparative Transcriptomic Study Reveals Temporal and Genotype-Specific Defense Responses to Botrytis cinerea in Grapevine
Source: J Fungi (Basel). 2025 Feb 7;11(2):124. doi: 10.3390/jof11020124 (PMC11856255; doi:10.3390/jof11020124)
Supplement: Supplementary file 1 [file jof-11-00124-s001.zip › FigureS10.pdf]

STILBENOID, DIARYLHEPTANOID AND GINGEROL BIOSYNTHESIS

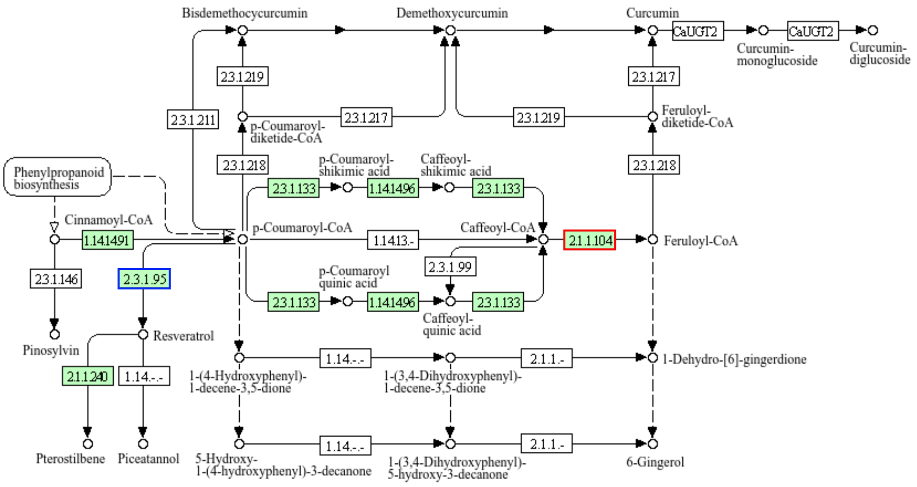

Tolerant genotype at T1 (IvsNI)

Down regulated genes 2.3.1.95:
Vitvi00g04407(-0.9341)
Vitvi00g04588(-1.3860)
Vitvi16g04347(-0.6328)

Up regulated genes 2.1.1.104:
Vitvi07g01723(0.7023)

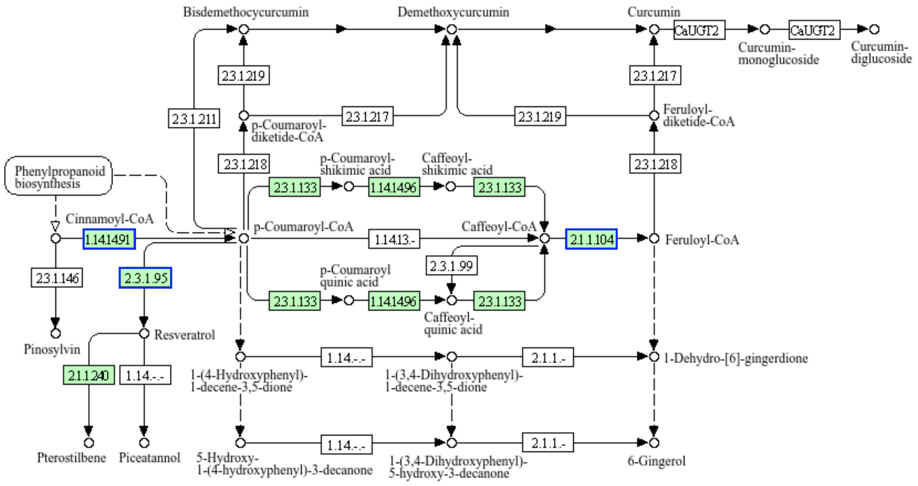

Tolerant genotype at T2 (IvsNI)

Down regulated genes 1.14.1491:
Vitvi11g00924(-1.9065)
Vitvi11g01045(-3.1018)
Down regulated genes 2.3.1.95:
Vitvi16g04353(-2.3965)
Vitvi16g04348(-2.2987)
Vitvi16g04347(-2.1230)
Vitvi00g04407(-2.0927)
Vitvi16g04349(-1.7950)
Vitvi16g01485(-1.3217)
Vitvi10g04623(-1.9301)
Vitvi00g04555(-1.8142)
Vitvi16g04345(-2.6040)
Down regulated genes 2.1.1.104:
Vitvi03g00524(-1.9922)

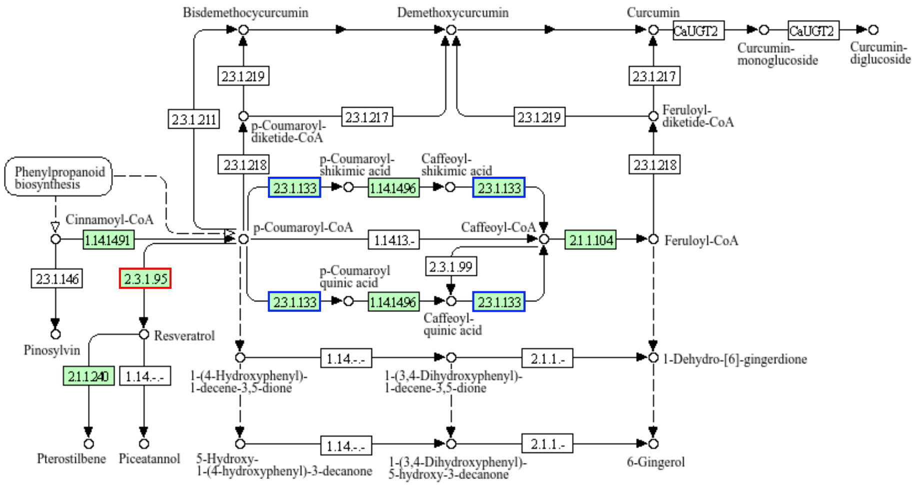

Susceptible genotype at T1 (IvsNI)

Down regulated genes 23.1.133:
Vitvi07g01255(-1.3354)

Up regulated genes 2.3.1.95:
Vitvi16g04351(2.4752)
Vitvi16g04349(1.6091)
Vitvi16g04347(1.9379)
Vitvi16g04348(2.2414)
Vitvi10g04623(1.9243)
Vitvi00g04555(2.6228)
Vitvi16g01485(1.3500)
Vitvi00g04407(1.2659)
Vitvi10g04624(1.9485)
Vitvi00g04588(1.4105)
Vitvi16g04346(2.6229)
Vitvi16g04345(1.9909)

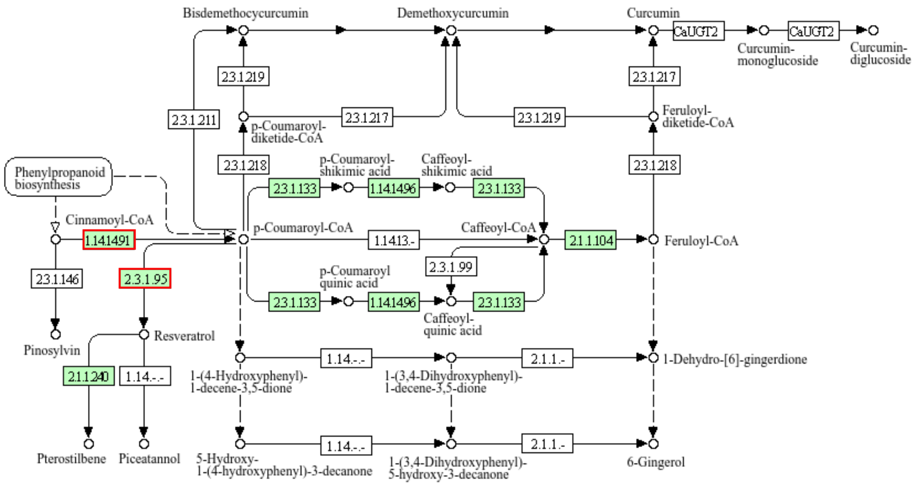

Susceptible genotype at T2 (IvsNI)

Up regulated genes 1.14.1491:
Vitvi11g00924(1.9120)
Vitvi06g00803(1.2520)
Up regulated genes 2.3.1.95:
Vitvi16g04349(1.556)
Vitvi00g04555(3.7135)
Vitvi10g04623(2.6353)
Vitvi00g04407(1.6020)
Vitvi00g04588(2.6222)
Vitvi16g04351(2.5901)
Vitvi16g04350(6.6970)
Vitvi16g04348(1.2873)
Vitvi16g04347(1.5253)
Vitvi16g04346(3.1731)
