# Supplementary material for: A Comparative Transcriptomic Study Reveals Temporal and Genotype-Specific Defense Responses to Botrytis cinerea in Grapevine
Source: J Fungi (Basel). 2025 Feb 7;11(2):124. doi: 10.3390/jof11020124 (PMC11856255; doi:10.3390/jof11020124)
Supplement: Supplementary file 1 [file jof-11-00124-s001.zip › FigureS11.pdf]

MAPK SIGNALING PATHWAY

Tolerant genotype at T1 (IvsNI)

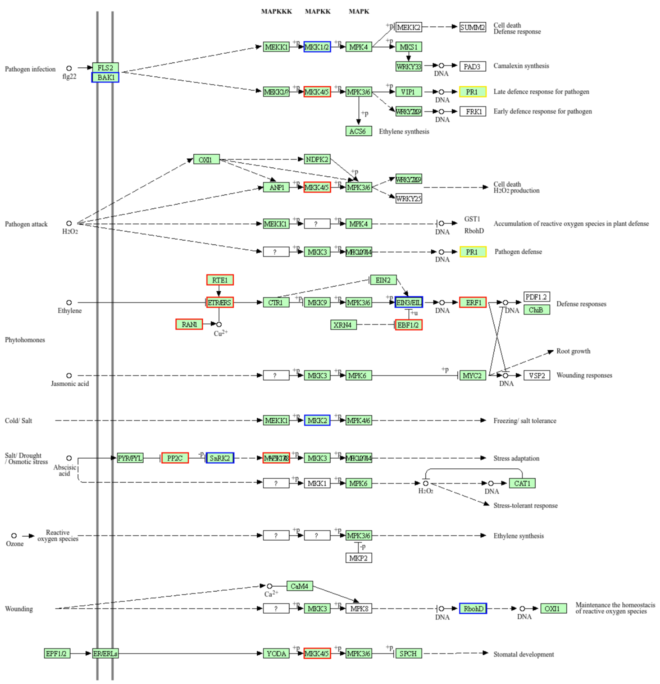

Down regulated genes BAK1
Vitvi12g01323(-1.1557)
Down regulated genes MKK1/2
Vitvi11g00136(-0.5621)
Down regulated genes PR1
Vitvi03g01651(-2.0354)
Down regulated genes EIN3/EIL
Vitvi06g01036(-0.6236)
Down regulated genes SnRK2
Vitvi07g01323(-0.7358)
Down regulated genes RbohD
Vitvi01g01803(-0.8245)

Up regulated genes MKK4/5
Vitvi09g01287(0.6106)
Up regulated genes PR1
Vitvi03g01649(4.3685)
Up regulated genes RTE1
Vitvi04g00115(0.4708)
Up regulated genes ETR/ERS
Vitvi07g00359(0.3360)
Up regulated genes RAN1
Vitvi02g01382(0.9380)
Up regulated genes EBF1/2
Vitvi11g00475(0.4624)
Up regulated genes ERF1
Vitvi07g02067(0.6993)
Up regulated genes PP2C
Vitvi13g00344(0.7601)
Vitvi06g00533(0.9044)
Vitvi02g00126(4.6437)
Up regulated genes MAPK17/18
Vitvi19g00528(2.5308)

Tolerant genotype at T2 (IvsNI)

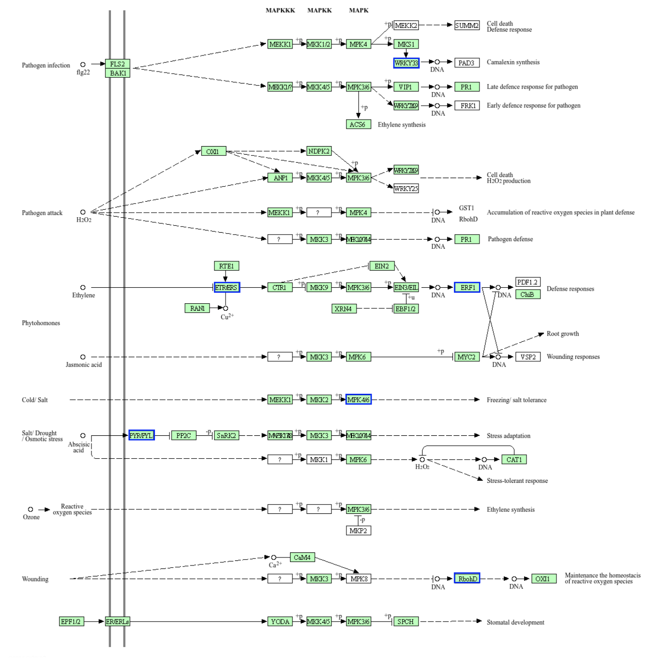

Down regulated genes MAPK4:
Vitvi15g01077(-0.4622)
Down regulated genes WRKY33:
Vitvi06g00741(-0.6624)
Vitvi08g00793(-1.0730)
Down regulated genes ACS6:
Vitvi02g00032(-3.8507)
Vitvi15g01093(-1.3456)
Down regulated genes WRKY22/29
Vitvi15g01090(-1.5673)
Vitvi02g00039(-1.3984)
Down regulated genes ETR/ERS:
Vitvi05g00684(-0.6013416)
Down regulated genes ERF1:
Vitvi05g00715(-1.6487)
Down regulated genes MPK4/6:
Vitvi15g01077(-0.4622)
Down regulated genes PYR/PYL:
Vitvi13g00114(-4.0323)
Down regulated genes RbohD:
Vitvi01g01803(-1.3919)
Vitvi14g00183(-1.7716)

Susceptible genotype at T1 (IvsNI)

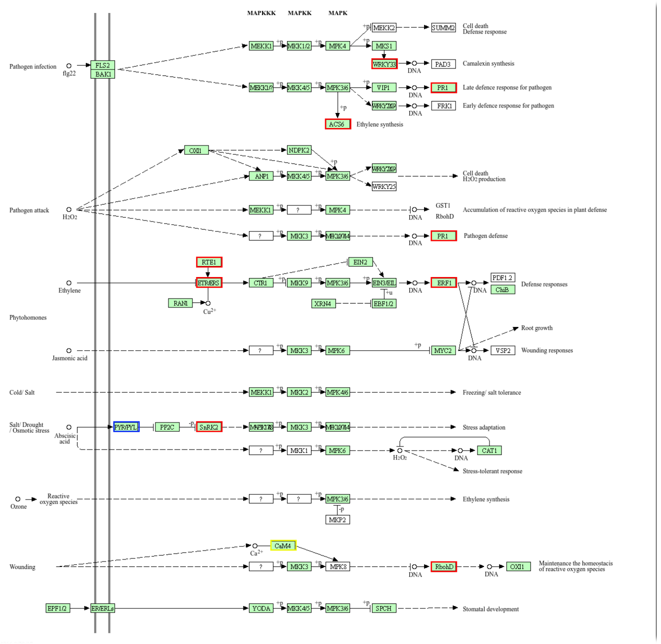

Down regulated genes PYR/PYL:
Vitvi15g00997(-0.6970)
Down regulated genes CaM4:
Vitvi05g01776(-0.9509)
Vitvi08g04152(-0.4088)

Up regulated genes WRKY33:
Vitvi08g00793(1.0651)
Up regulated genes PR1:
Vitvi03g01650(1.5921)
Up regulated genes ACS6:
Vitvi02g00032(2.7005)
Up regulated genes RTE1:
Vitvi04g00115(0.8233)
Up regulated genesETR/ERF:
Vitvi05g00684(0.7245)
Up regulated genes ERF1:
Vitvi05g00715(1.8203)
Up regulated genes SnRK2:
Vitvi18g00440(0.5574)
Up regulated genes CaM4:
Vitvi07g00068(1.6367)
Up regulated genes RbohD:
Vitvi14g00183(1.3627)
Vitvi01g01803(1.0870)

Susceptible genotype at T2 (IvsNI)

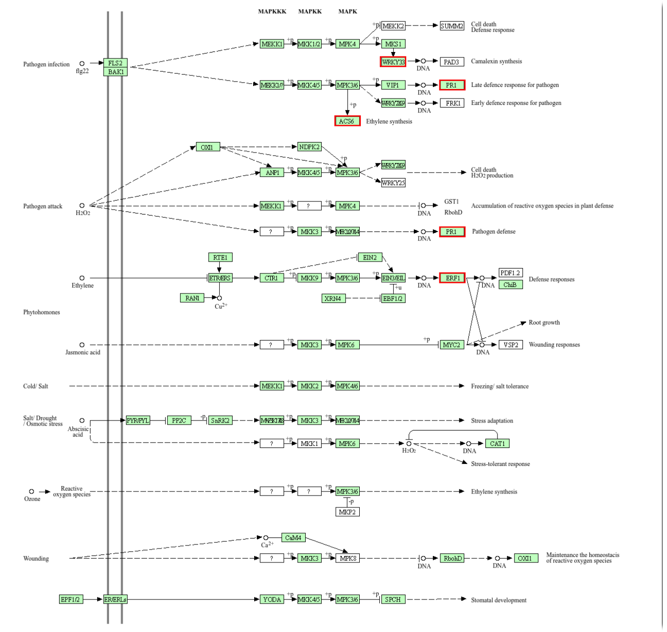

Up regulated genes WRKY33:
Vitvi08g00793(1.1551)
Up regulated genes PR1:
Vitvi03g01650(1.8625)
Up regulated genes ACS6:
Vitvi02g00032(8.0744)
Up regulated genes ERF1:
Vitvi05g00715(4.4709)
