# Supplementary material for: A Comparative Transcriptomic Study Reveals Temporal and Genotype-Specific Defense Responses to Botrytis cinerea in Grapevine
Source: J Fungi (Basel). 2025 Feb 7;11(2):124. doi: 10.3390/jof11020124 (PMC11856255; doi:10.3390/jof11020124)
Supplement: Supplementary file 1 [file jof-11-00124-s001.zip › FigureS6.pdf]

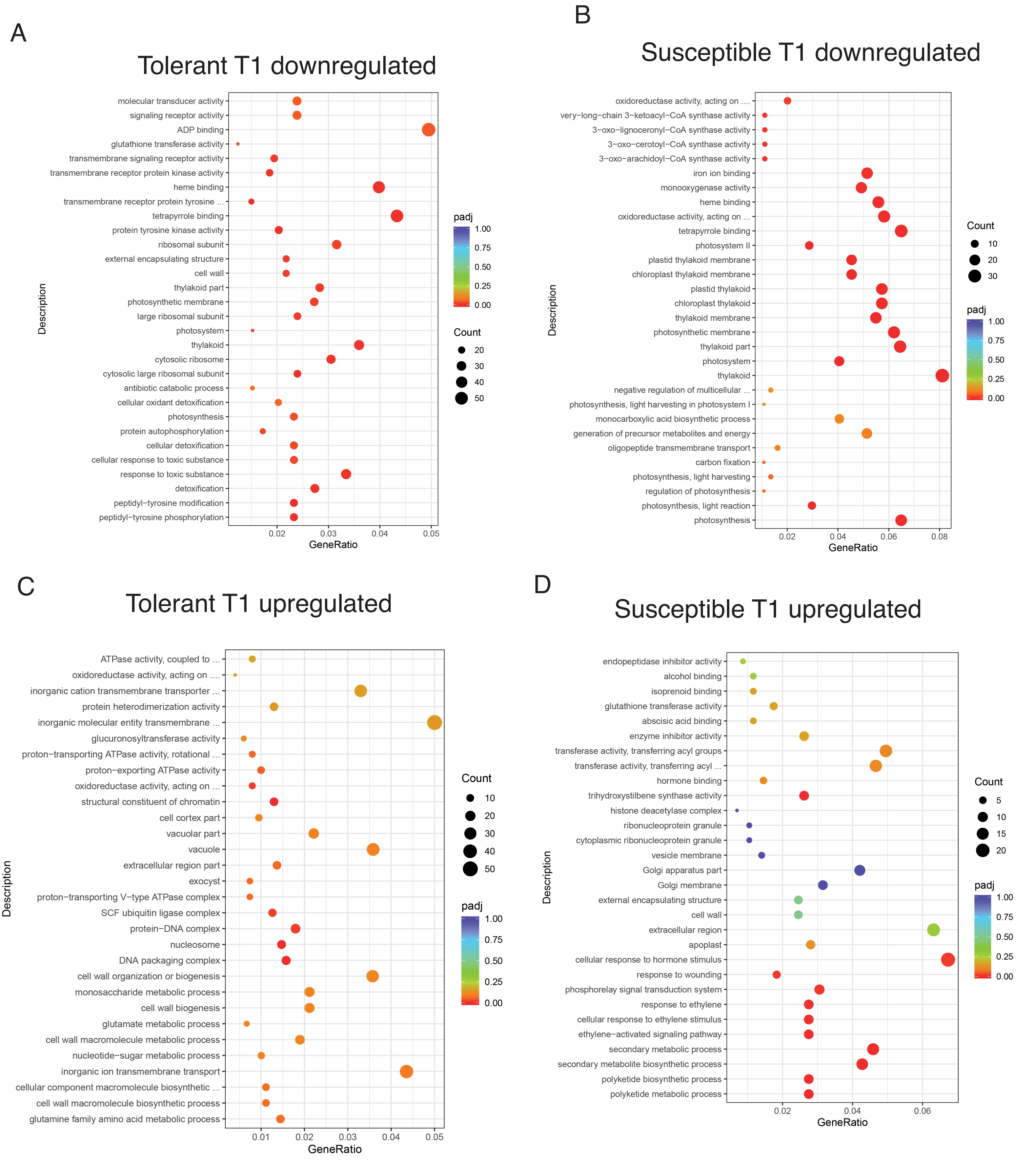

A
Tolerant T1 downregulated
molecular transducer activity
signaling receptor activity
ADP binding
glutathione transferase activity
transmembrane signaling receptor activity
transmembrane receptor protein kinase activity
heme binding
transmembrane receptor protein tyrosine ...
tetrapyrrole binding
protein tyrosine kinase activity
ribosomal subunit
external encapsulating structure
cell wall
thylakoid part
photosynthetic membrane
large ribosomal subunit
photosystem
thylakoid
cytosolic ribosome
cytosolic large ribosomal subunit
antibiotic catabolic process
cellular oxidant detoxification
photosynthesis
protein autophosphorylation
cellular detoxification
cellular response to toxic substance
response to toxic substance
detoxification
peptidyl−tyrosine modification
peptidyl−tyrosine phosphorylation
Description
GeneRatio
0.02 0.03 0.04 0.05
padj
1.00 0.75 0.50 0.25 0.00
Count
20 30 40 50
B
Susceptible T1 downregulated
oxidoreductase activity, acting on ....
very−long−chain 3−ketoacyl−CoA synthase activity
3−oxo−lignoceronyl−CoA synthase activity
3−oxo−cerotoyl−CoA synthase activity
3−oxo−arachidoyl−CoA synthase activity
iron ion binding
monooxygenase activity
heme binding
oxidoreductase activity, acting on ...
tetrapyrrole binding
photosystem II
plastid thylakoid membrane
chloroplast thylakoid membrane
plastid thylakoid
chloroplast thylakoid
thylakoid membrane
photosynthetic membrane
thylakoid part
photosystem
thylakoid
negative regulation of multicellular ...
photosynthesis, light harvesting in photosystem I
monocarboxylic acid biosynthetic process
generation of precursor metabolites and energy
oligopeptide transmembrane transport
carbon fixation
photosynthesis, light harvesting
regulation of photosynthesis
photosynthesis, light reaction
photosynthesis
Description
GeneRatio
0.02 0.04 0.06 0.08
Count
10 20 30
padj
1.00 0.75 0.50 0.25 0.00
C
Tolerant T1 upregulated
ATPase activity, coupled to ...
oxidoreductase activity, acting on ....
inorganic cation transmembrane transporter ...
protein heterodimerization activity
inorganic molecular entity transmembrane ...
glucuronosyltransferase activity
proton−transporting ATPase activity, rotational ...
proton−exporting ATPase activity
oxidoreductase activity, acting on ...
structural constituent of chromatin
cell cortex part
vacuolar part
vacuole
extracellular region part
exocyst
proton−transporting V−type ATPase complex
SCF ubiquitin ligase complex
protein−DNA complex
nucleosome
DNA packaging complex
cell wall organization or biogenesis
monosaccharide metabolic process
cell wall biogenesis
glutamate metabolic process
cell wall macromolecule metabolic process
nucleotide−sugar metabolic process
inorganic ion transmembrane transport
cellular component macromolecule biosynthetic ...
cell wall macromolecule biosynthetic process
glutamine family amino acid metabolic process
Description
GeneRatio
0.01 0.02 0.03 0.04 0.05
Count
10 20 30 40 50
padj
1.00 0.75 0.50 0.25 0.00
D
Susceptible T1 upregulated
endopeptidase inhibitor activity
alcohol binding
isoprenoid binding
glutathione transferase activity
abscisic acid binding
enzyme inhibitor activity
transferase activity, transferring acyl groups
transferase activity, transferring acyl ...
hormone binding
trihydroxystilbene synthase activity
histone deacetylase complex
ribonucleoprotein granule
cytoplasmic ribonucleoprotein granule
vesicle membrane
Golgi apparatus part
Golgi membrane
external encapsulating structure
cell wall
extracellular region
apoplast
cellular response to hormone stimulus
response to wounding
phosphorelay signal transduction system
response to ethylene
cellular response to ethylene stimulus
ethylene−activated signaling pathway
secondary metabolic process
secondary metabolite biosynthetic process
polyketide biosynthetic process
polyketide metabolic process
Description
GeneRatio
0.02 0.04 0.06
Count
5 10 15 20
padj
1.00 0.75 0.50 0.25 0.00
