# Supplementary material for: A Comparative Transcriptomic Study Reveals Temporal and Genotype-Specific Defense Responses to Botrytis cinerea in Grapevine
Source: J Fungi (Basel). 2025 Feb 7;11(2):124. doi: 10.3390/jof11020124 (PMC11856255; doi:10.3390/jof11020124)
Supplement: Supplementary file 1 [file jof-11-00124-s001.zip › FigureS8.pdf]

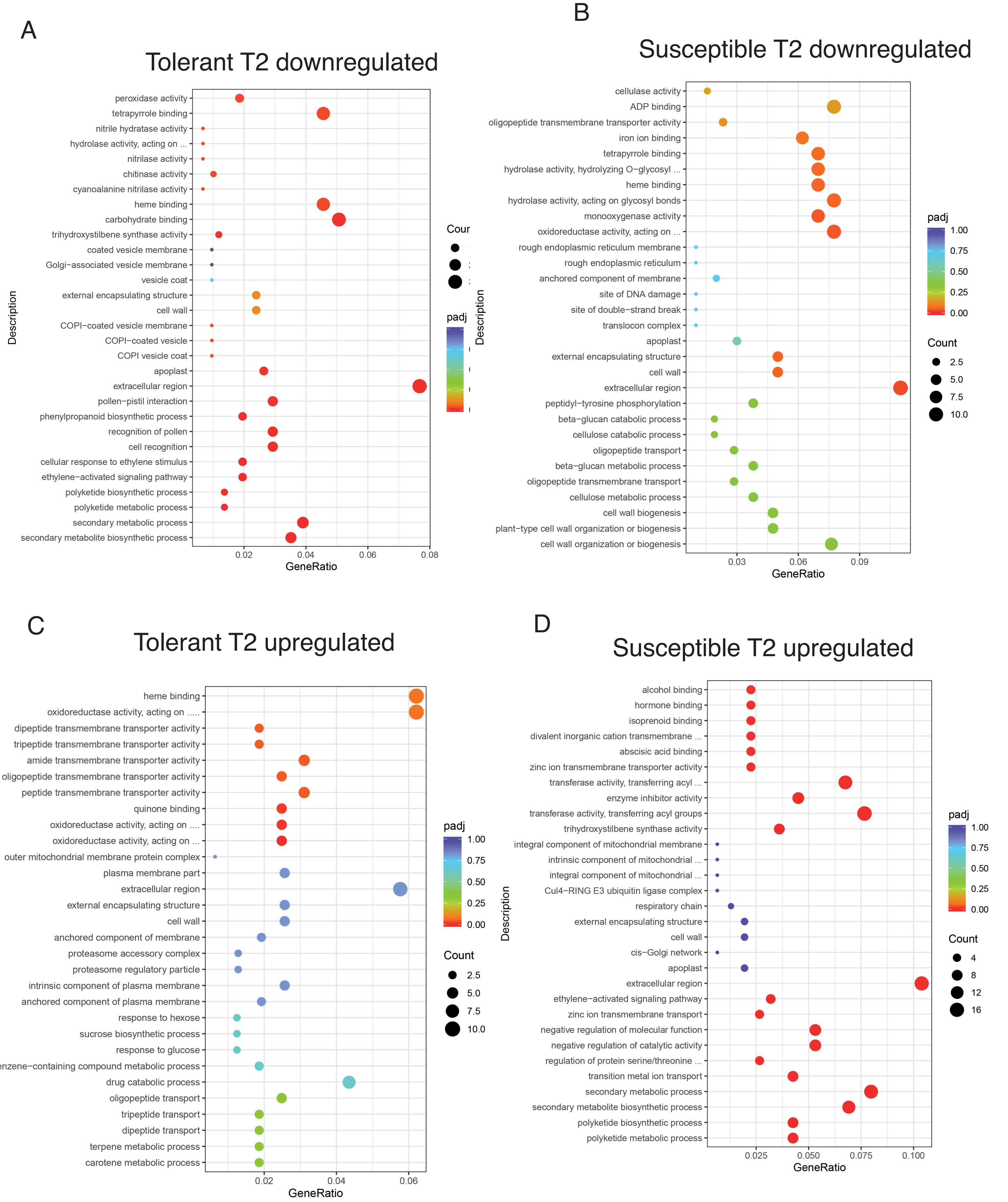
A
Tolerant T2 downregulated
peroxidase activity
tetrapyrrole binding
nitrile hydratase activity
hydrolase activity, acting on ...
nitrilase activity
chitinase activity
cyanoalanine nitrilase activity
heme binding
carbohydrate binding
trihydroxystilbene synthase activity
coated vesicle membrane
Golgi−associated vesicle membrane
vesicle coat
external encapsulating structure
cell wall
COPI−coated vesicle membrane
COPI−coated vesicle
COPI vesicle coat
apoplast
extracellular region
pollen−pistil interaction
phenylpropanoid biosynthetic process
recognition of pollen
cell recognition
cellular response to ethylene stimulus
ethylene−activated signaling pathway
polyketide biosynthetic process
polyketide metabolic process
secondary metabolic process
secondary metabolite biosynthetic process
Description
0.02
0.04
0.06
0.08
GeneRatio
Cour
padj
B
Susceptible T2 downregulated
cellulase activity
ADP binding
oligopeptide transmembrane transporter activity
iron ion binding
tetrapyrrole binding
hydrolase activity, hydrolyzing O−glycosyl ...
heme binding
hydrolase activity, acting on glycosyl bonds
monooxygenase activity
oxidoreductase activity, acting on ...
rough endoplasmic reticulum membrane
rough endoplasmic reticulum
anchored component of membrane
site of DNA damage
site of double−strand break
translocon complex
apoplast
external encapsulating structure
cell wall
extracellular region
peptidyl−tyrosine phosphorylation
beta−glucan catabolic process
cellulose catabolic process
oligopeptide transport
beta−glucan metabolic process
oligopeptide transmembrane transport
cellulose metabolic process
cell wall biogenesis
plant−type cell wall organization or biogenesis
cell wall organization or biogenesis
Description
0.03
0.06
0.09
GeneRatio
padj
1.00
0.75
0.50
0.25
0.00
Count
2.5
5.0
7.5
10.0
C
Tolerant T2 upregulated
heme binding
oxidoreductase activity, acting on .....
dipeptide transmembrane transporter activity
tripeptide transmembrane transporter activity
amide transmembrane transporter activity
oligopeptide transmembrane transporter activity
peptide transmembrane transporter activity
quinone binding
oxidoreductase activity, acting on ....
oxidoreductase activity, acting on ...
outer mitochondrial membrane protein complex
plasma membrane part
extracellular region
external encapsulating structure
cell wall
anchored component of membrane
proteasome accessory complex
proteasome regulatory particle
intrinsic component of plasma membrane
anchored component of plasma membrane
response to hexose
sucrose biosynthetic process
response to glucose
benzene−containing compound metabolic process
drug catabolic process
oligopeptide transport
tripeptide transport
dipeptide transport
terpene metabolic process
carotene metabolic process
Description
0.02
0.04
0.06
GeneRatio
padj
1.00
0.75
0.50
0.25
0.00
Count
2.5
5.0
7.5
10.0
D
Susceptible T2 upregulated
alcohol binding
hormone binding
isoprenoid binding
divalent inorganic cation transmembrane ...
abscisic acid binding
zinc ion transmembrane transporter activity
transferase activity, transferring acyl ...
enzyme inhibitor activity
transferase activity, transferring acyl groups
trihydroxystilbene synthase activity
integral component of mitochondrial membrane
intrinsic component of mitochondrial ...
integral component of mitochondrial ...
Cul4−RING E3 ubiquitin ligase complex
respiratory chain
external encapsulating structure
cell wall
cis−Golgi network
apoplast
extracellular region
ethylene−activated signaling pathway
zinc ion transmembrane transport
negative regulation of molecular function
negative regulation of catalytic activity
regulation of protein serine/threonine ...
transition metal ion transport
secondary metabolic process
secondary metabolite biosynthetic process
polyketide biosynthetic process
polyketide metabolic process
Description
0.025
0.050
0.075
0.100
GeneRatio
padj
1.00
0.75
0.50
0.25
0.00
Count
4
8
12
16
