# Supplementary figures and images for: A Comparative Transcriptomic Study Reveals Temporal and Genotype-Specific Defense Responses to Botrytis cinerea in Grapevine
Source: J Fungi (Basel). 2025 Feb 7;11(2):124. doi: 10.3390/jof11020124 (PMC11856255; doi:10.3390/jof11020124)

A

Categories of the OIV descriptor n. 459

1-3 (very little to little)

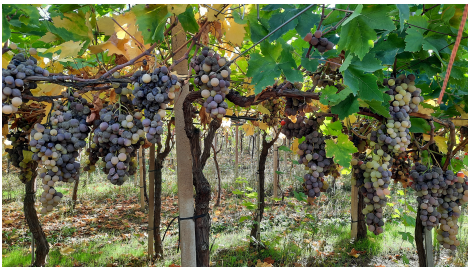

5 (medium)

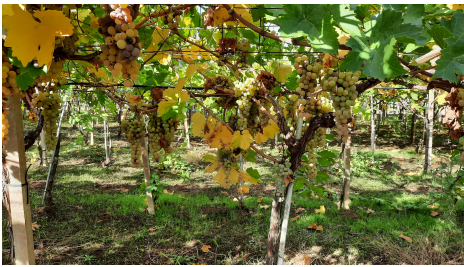

7-9 (high to very high)

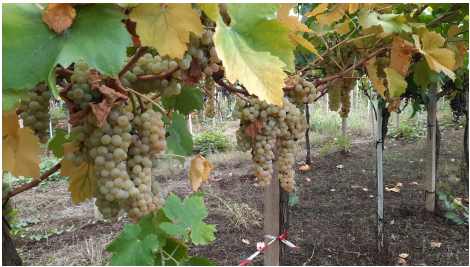

B

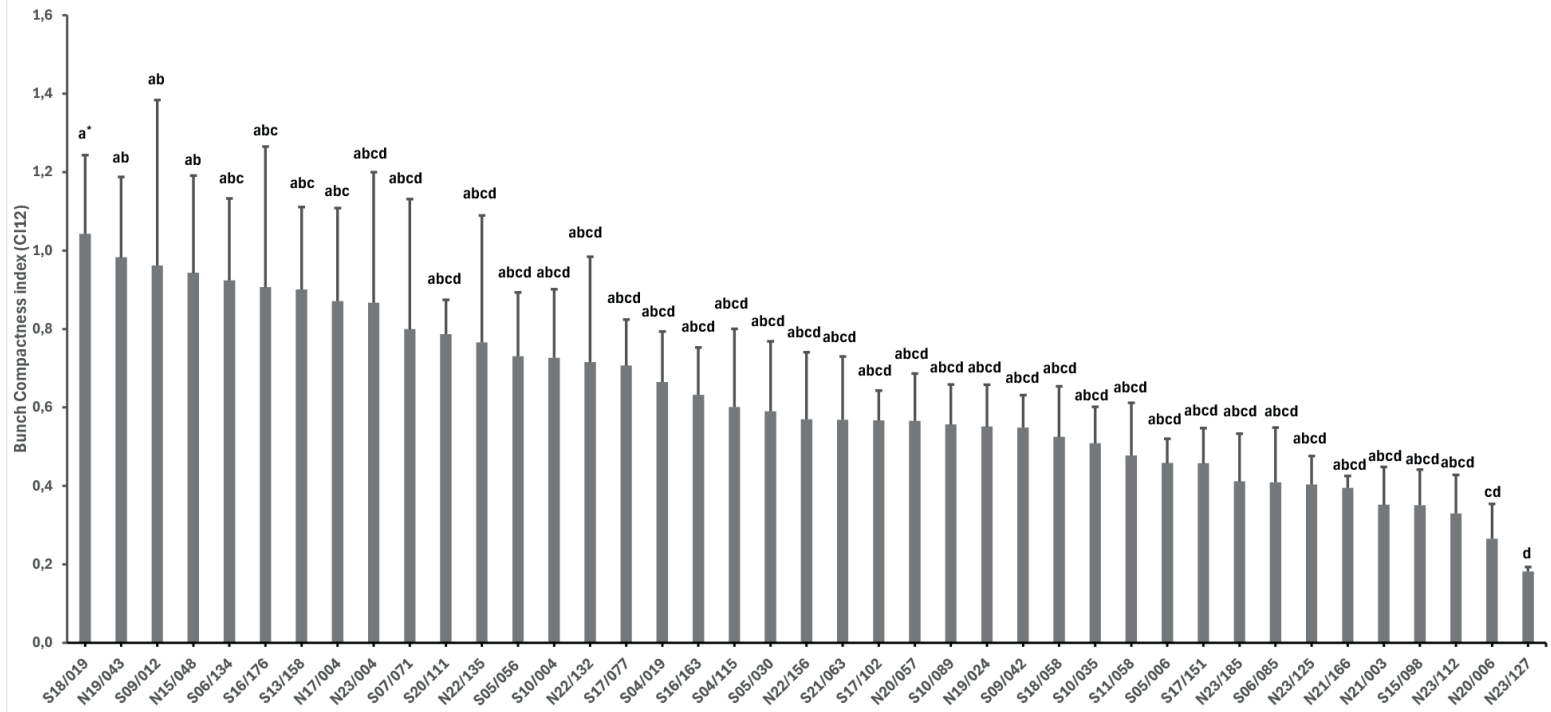

Supplement: Supplementary file 1 [file jof-11-00124-s001.zip › FigureS1.pdf]

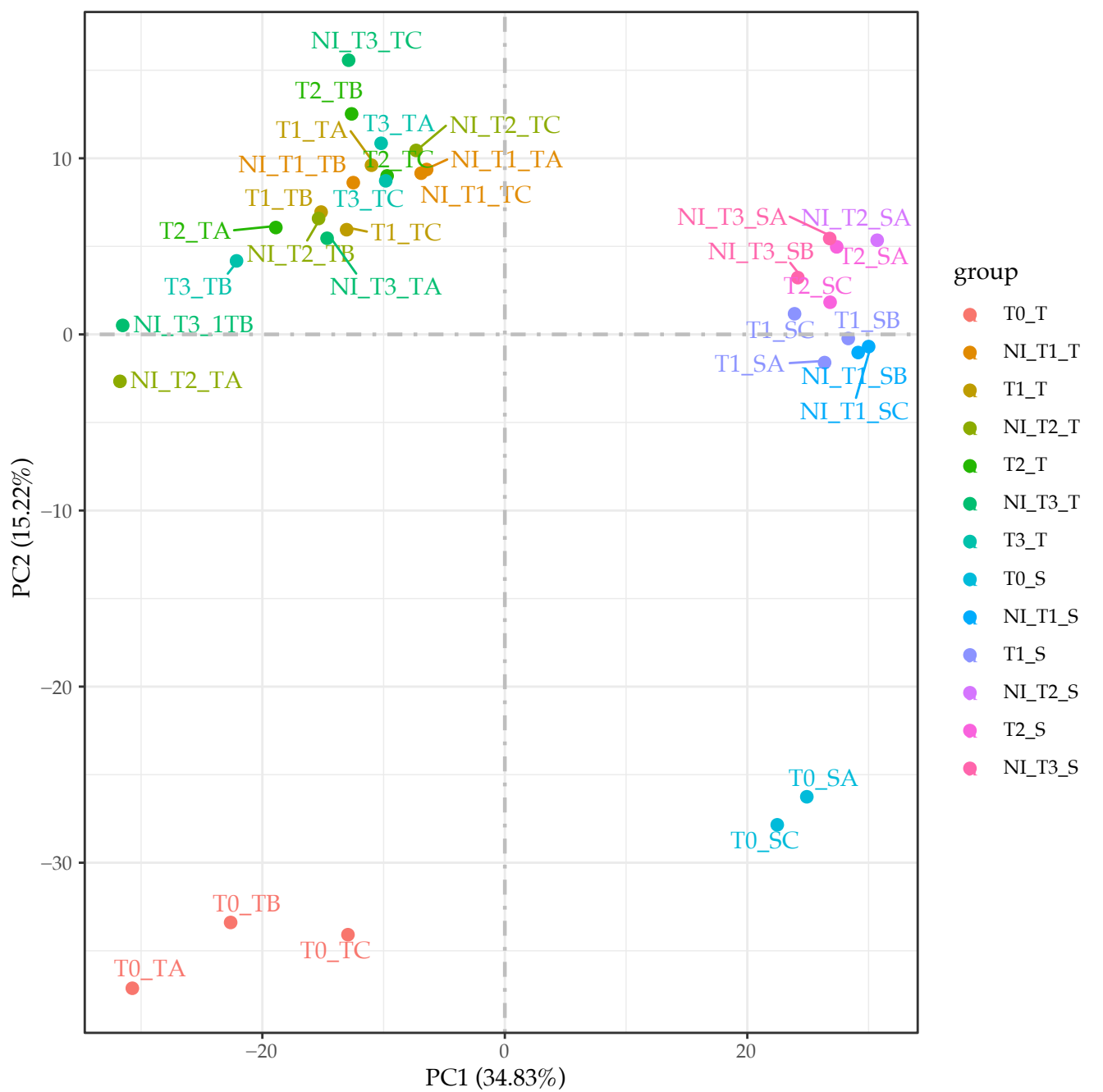

Supplement: Supplementary file 1 [file jof-11-00124-s001.zip › FigureS2.pdf]

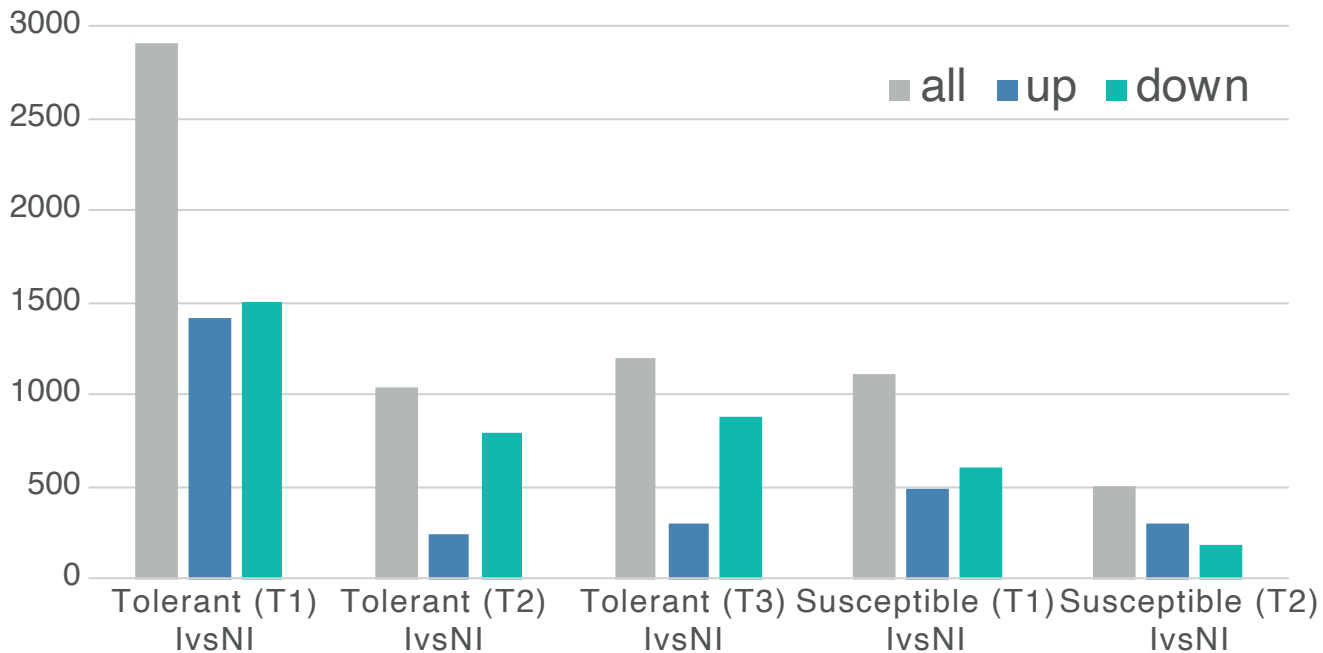

Supplement: Supplementary file 1 [file jof-11-00124-s001.zip › FigureS3.pdf]

A

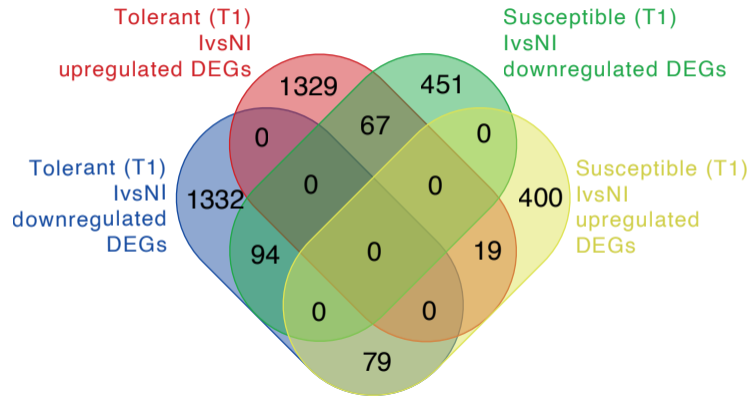

B

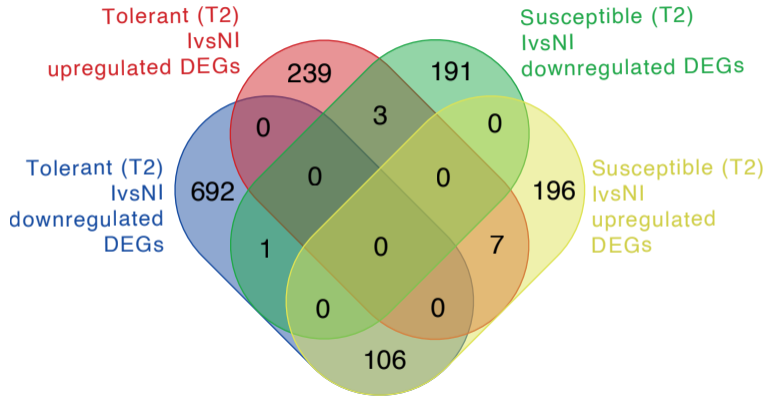

Supplement: Supplementary file 1 [file jof-11-00124-s001.zip › FigureS4.pdf]

A

Tolerant Genotype

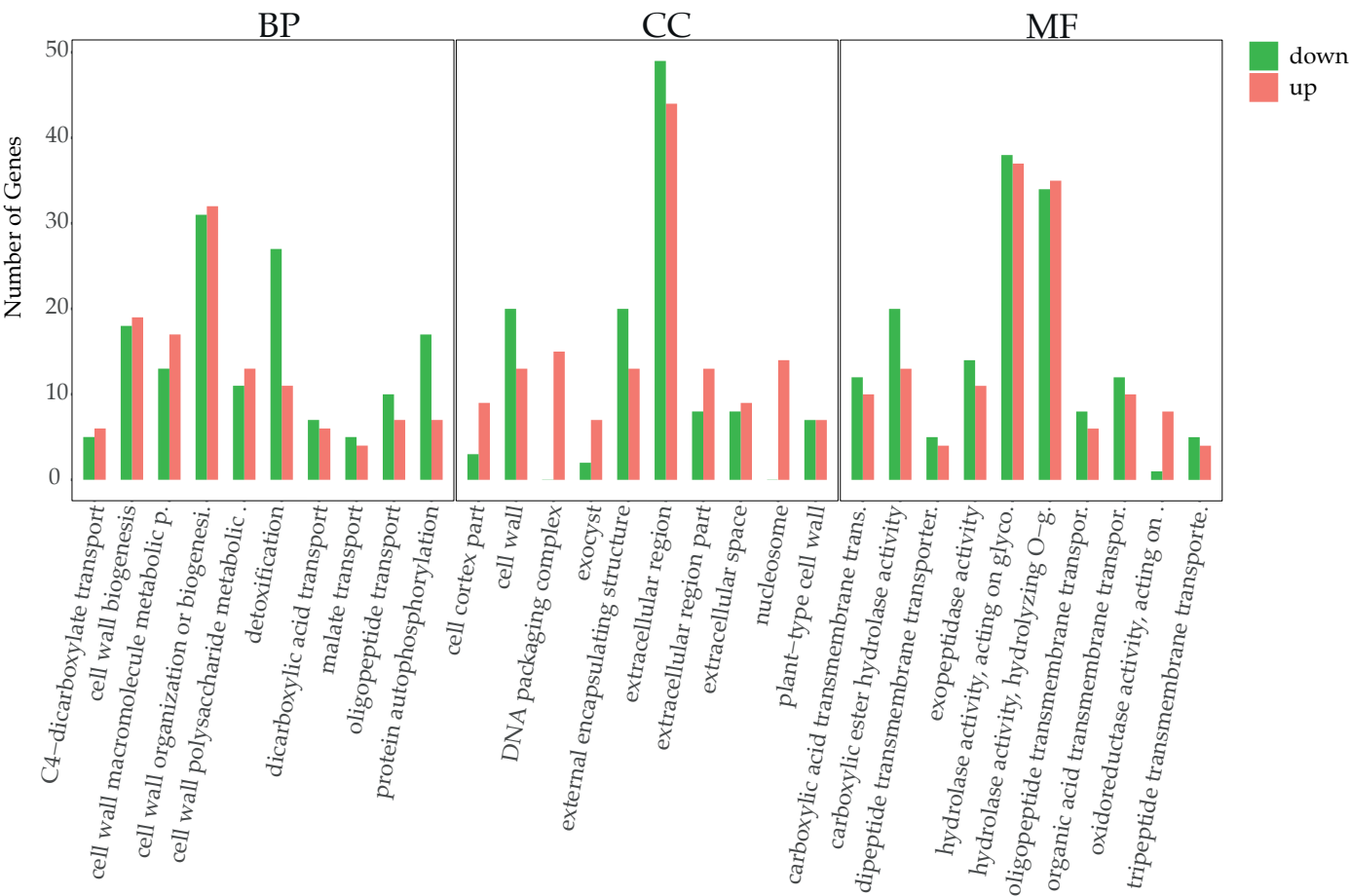

B

Susceptible Genotype

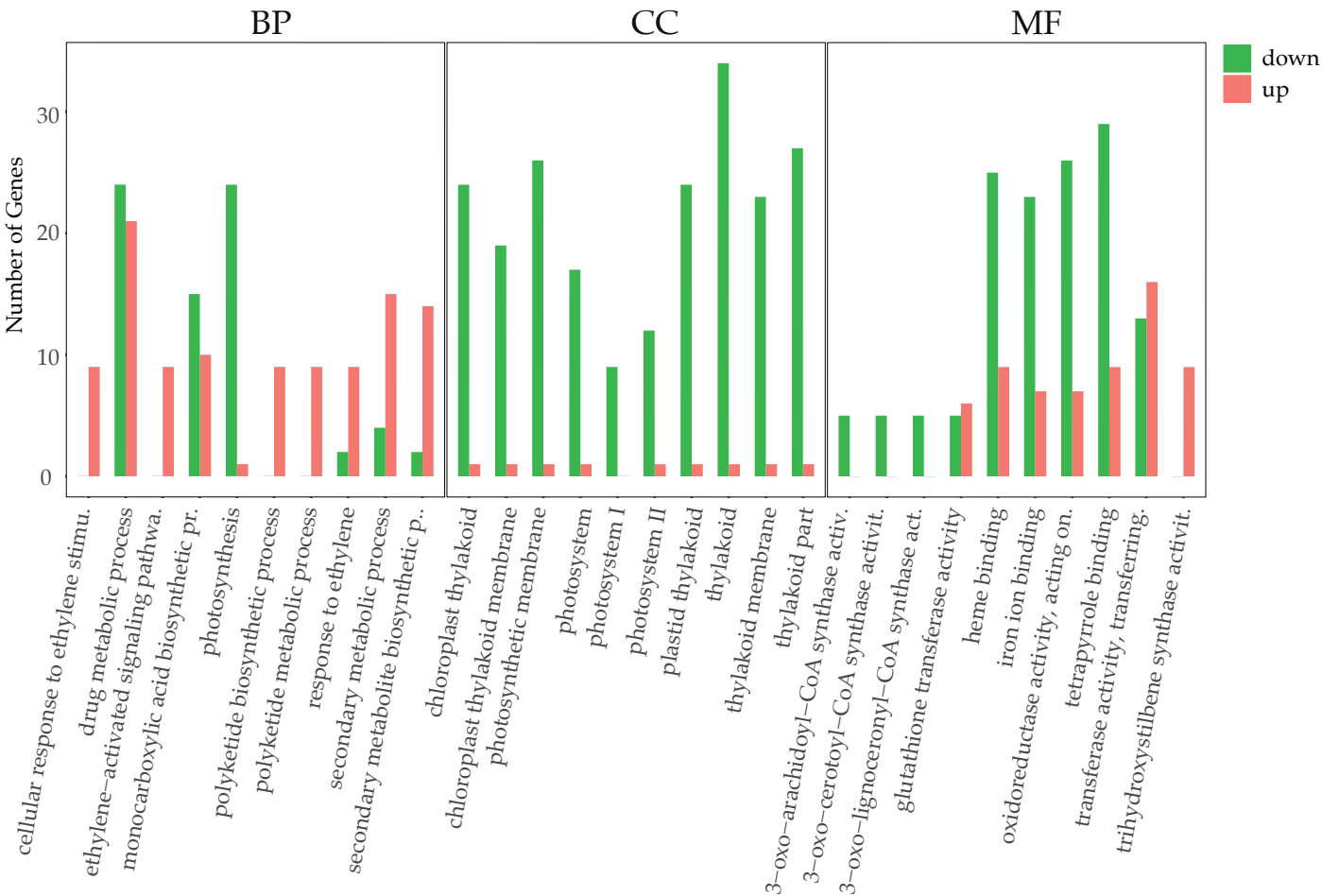

Supplement: Supplementary file 1 [file jof-11-00124-s001.zip › FigureS5.pdf]

A

## Tolerant Genotype

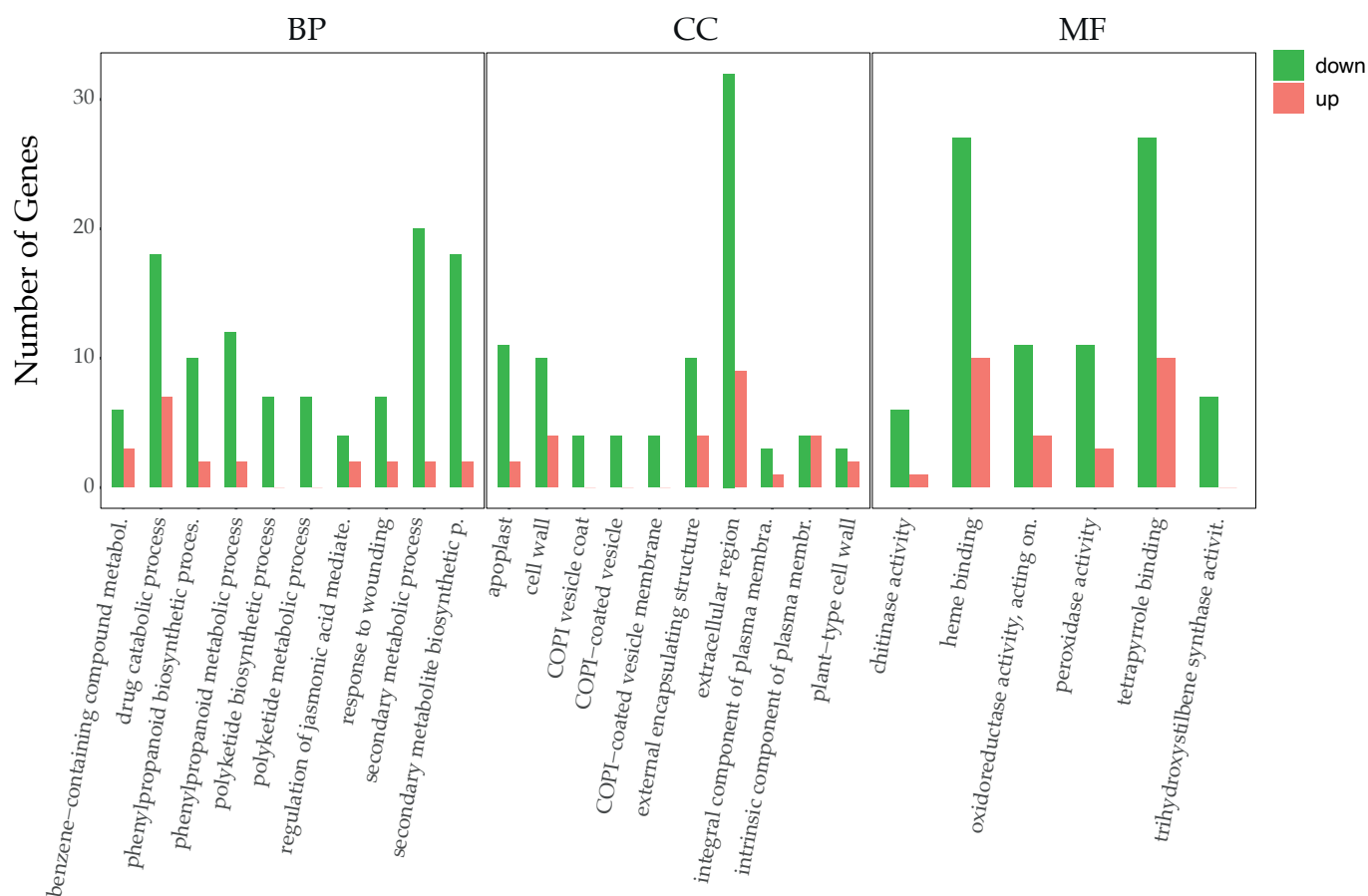

B

## Susceptible Genotype

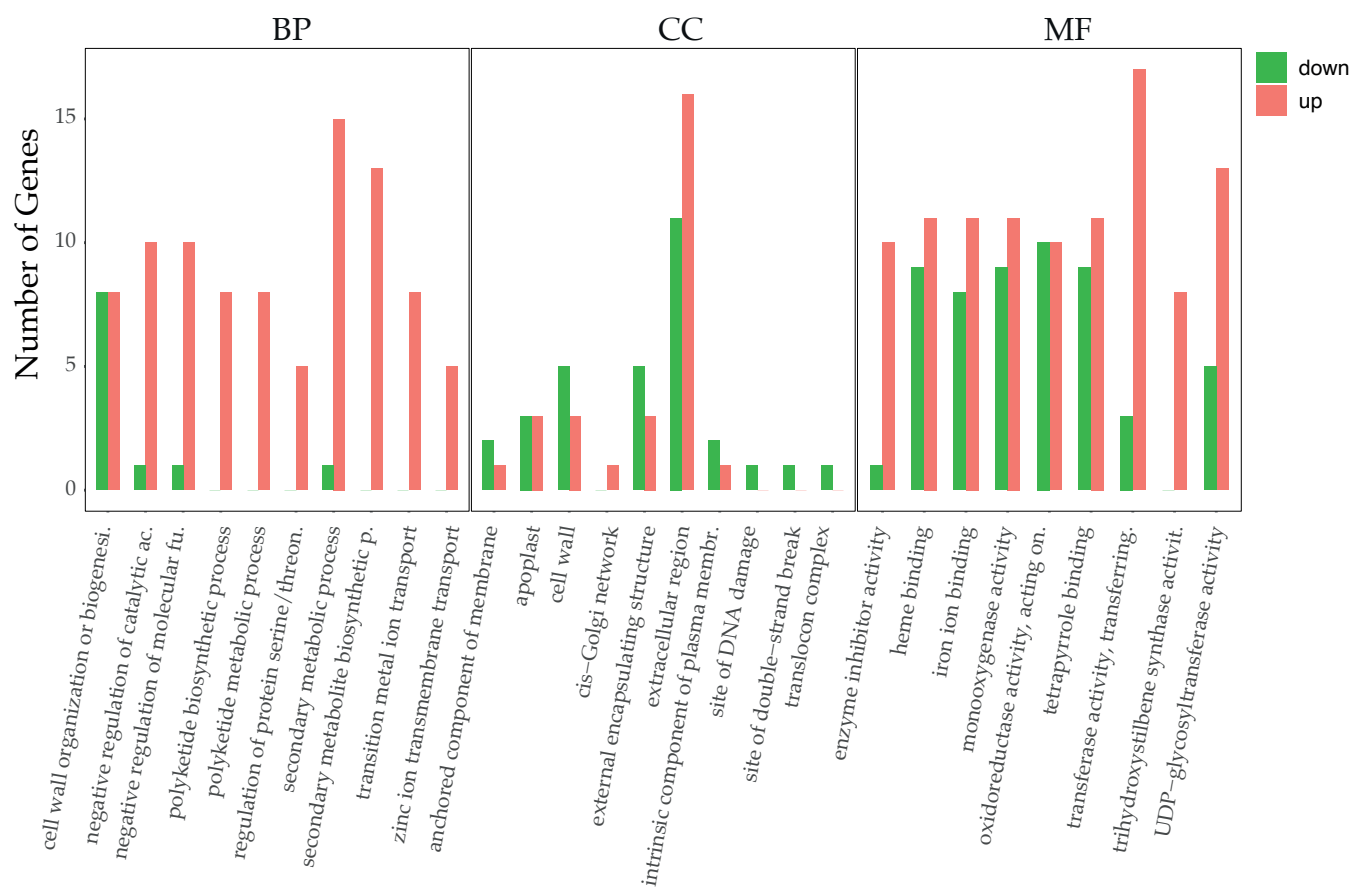

Supplement: Supplementary file 1 [file jof-11-00124-s001.zip › FigureS7.pdf]

A

## Tolerant T3 downregulated

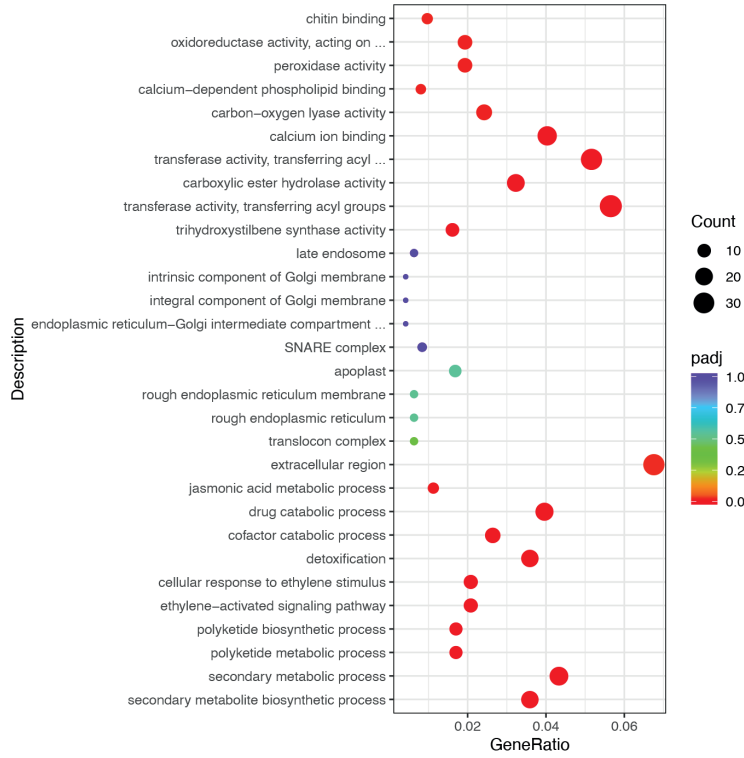

B

## Tolerant T3 upregulated

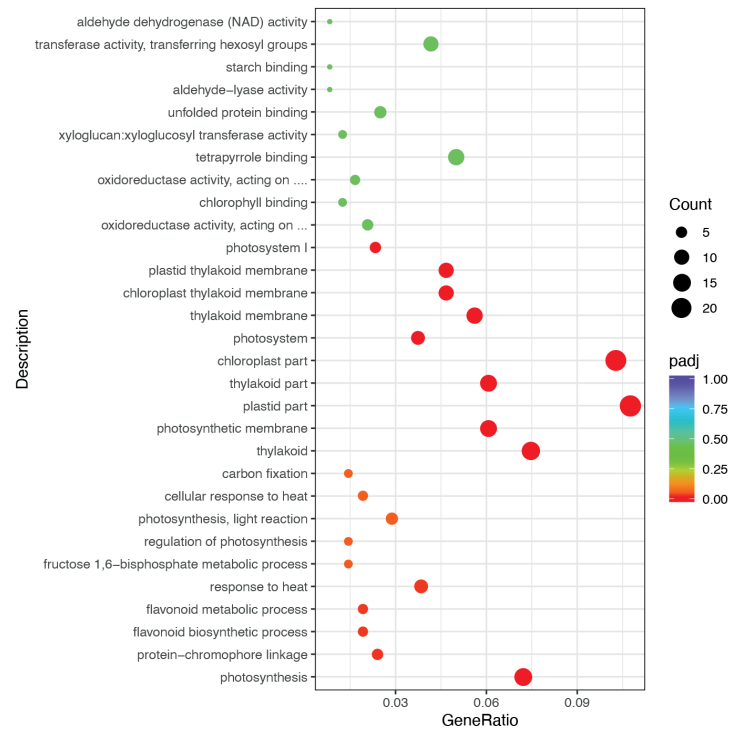

Supplement: Supplementary file 1 [file jof-11-00124-s001.zip › FigureS9.pdf]
